# *De Novo VPS4A* Mutations Cause Multisystem Disease with Abnormal Neurodevelopment

**DOI:** 10.1016/j.ajhg.2020.10.012

**Published:** 2020-11-12

**Authors:** Catherine Rodger, Elisabetta Flex, Rachel J. Allison, Alba Sanchis-Juan, Marcia A. Hasenahuer, Serena Cecchetti, Courtney E. French, James R. Edgar, Giovanna Carpentieri, Andrea Ciolfi, Francesca Pantaleoni, Alessandro Bruselles, Genomics England Research Consortium, Roberta Onesimo, Giuseppe Zampino, Francesca Marcon, Ester Siniscalchi, Melissa Lees, Deepa Krishnakumar, Emma McCann, Dragana Yosifova, Joanna Jarvis, Michael C. Kruer, Warren Marks, Jonathan Campbell, Louise E. Allen, Stefano Gustincich, F. Lucy Raymond, Marco Tartaglia, Evan Reid

**Affiliations:** 1 Cambridge Institute for Medical Research, University of Cambridge, Cambridge CB2 0XY, UK; 2 Department of Medical Genetics, University of Cambridge, Cambridge CB2 0QQ, UK; 3 Department of Oncology and Molecular Medicine, Istituto Superiore di Sanità, Rome 00161, Italy; 4 Department of Haematology, NHS Blood and Transplant Centre, University of Cambridge, Cambridge CB2 0XY, UK; 5 NIHR BioResource, Cambridge University Hospitals NHS Foundation Trust, Cambridge Biomedical Campus, Cambridge CB2 0QQ, UK; 6 European Molecular Biology Laboratory – European Bioinformatics Institute (EMBL-EBI), Wellcome Genome Campus, Hinxton, Cambridgeshire CB10 1SA, UK; 7 Microscopy Area, Core Facilities, Istituto Superiore di Sanità, Rome 00161, Italy; 8 Department of Pathology, University of Cambridge, Cambridge CB2 1QP, UK; 9 Genetics and Rare Diseases Research Division, Ospedale Pediatrico Bambino Gesù, IRCCS, Rome 00146, Italy; 10 Genomics England, London, UK; 11 Fondazione Policlinico Universitario A. Gemelli-IRCCS, Rome 00168, Italy; 12 Università Cattolica del Sacro Cuore, Rome 00168, Italy; 13 Unit of Mechanisms, Biomarkers and Models, Department of Environment and Health, Istituto Superiore di Sanità, Rome 00161, Italy; 14 Department of Clinical Genetics, Great Ormond Street Hospital, London WC1N 3JH, UK; 15 Department of Paediatric Neurology, Cambridge University Hospitals NHS Foundation Trust, Cambridge CB2 0QQ, UK; 16 Department of Clinical Genetics, Liverpool Women’s Hospital, Liverpool L8 7SS, UK; 17 Department of Medical Genetics, Guys’ and St Thomas’ NHS Foundation Trust, London SE1 9RT, UK; 18 Clinical Genetics, Birmingham Women’s and Children’s NHS Foundation Trust, Birmingham B15 2TG, UK; 19 Phoenix Children’s Hospital, Phoenix, AZ 76109, USA; 20 Cook Children’s Medical Centre, Fort Worth, TX 76104, USA; 21 Colchester Hospital, East Suffolk and North Essex NHS Foundation Trust, Essex CO4 5JL, UK; 22 Ophthalmology Department, Cambridge University Hospitals NHS Foundation Trust, Cambridge CB2 0QQ, UK; 23 Department of Neuroscience and Brain Technologies, Istituto Italiano di Tecnologia, Genova 16163, Italy; 24 Area of Neuroscience, SISSA, Trieste 34136, Italy

**Keywords:** endosomal sorting complex required for transport, microcephaly, endosomal sorting, mitosis, nuclear envelope, DNA damage, primary cilium, cerebellar hypoplasia, centrosome, CIMDAG

## Abstract

The endosomal sorting complexes required for transport (ESCRTs) are essential for multiple membrane modeling and membrane-independent cellular processes. Here we describe six unrelated individuals with *de novo* missense variants affecting the ATPase domain of VPS4A, a critical enzyme regulating ESCRT function. Probands had structural brain abnormalities, severe neurodevelopmental delay, cataracts, growth impairment, and anemia. In cultured cells, overexpression of VPS4A mutants caused enlarged endosomal vacuoles resembling those induced by expression of known dominant-negative ATPase-defective forms of VPS4A. Proband-derived fibroblasts had enlarged endosomal structures with abnormal accumulation of the ESCRT protein IST1 on the limiting membrane. VPS4A function was also required for normal endosomal morphology and IST1 localization in iPSC-derived human neurons. Mutations affected other ESCRT-dependent cellular processes, including regulation of centrosome number, primary cilium morphology, nuclear membrane morphology, chromosome segregation, mitotic spindle formation, and cell cycle progression. We thus characterize a distinct multisystem disorder caused by mutations affecting *VPS4A* and demonstrate that its normal function is required for multiple human developmental and cellular processes.

## Introduction

The endosomal sorting complexes required for transport (ESCRTs) are multifunctional membrane modeling machineries that drive membrane fission or constriction in cellular processes that involve “inside out” membrane topology.^1^, ^2^, ^3^ These are exemplified by fission reactions that cause vesicle budding away from the cytoplasm, in which ESCRT-III complexes assemble on the inner cytosolic face of a vesicle neck and promote membrane constriction from the inside. Modification of ESCRT-III complexes drives fission, and this is performed by the catalytic activity of members of the VPS4 ATPase family (which in vertebrates comprises two paralogs, VPS4A and VPS4B)—thus VPS4 is an indispensable component of all ESCRT-related membrane modeling.^4^ Processes that involve this type of membrane topology include formation of the late endosomal multivesicular body (MVB), nuclear envelope reformation, and the abscission stage of cell division, among others.^1^, ^2^, ^3^ In addition, certain ESCRT-III-associated proteins are active in more conventional “outside in” fission, notably in endosomal tubule fission, where atypical ESCRT-III proteins constrict from the outside to promote fission of sorting tubules from the endosomal body.^5^^,^^6^

Study of the ESCRT complexes and VPS4 in the endocytic pathway has informed mechanistic understanding of their role in membrane modeling.^2^^,^^3^^,^^7^, ^8^, ^9^ Endocytosis regulates the cell surface concentration of plasma membrane proteins and so controls multiple critical cellular processes. After endocytosis from the cell surface, membrane proteins are trafficked to the early sorting endosome, from where they may be sorted away from the endosomal system (e.g., to the plasma membrane) or retained for degradation in the late endosome-lysosome pathway.^10^ Membrane proteins to be degraded are exposed to the lumenal degradative compartment of the late endosome and lysosome by a process involving inward budding of the endosomal limiting membrane to form the intralumenal vesicles (ILVs) of the late endosome or multivesicular body (MVB). Concentration and sorting of cargoes into, and formation of, the ILVs is accomplished by the action of the ESCRT-0, I, II, and III complexes.^3^^,^^7^, ^8^, ^9^ In the final stage of this process, ILVs are released into the MVB lumen by the ESCRT-III complex, comprising a number of charged multivesicular body proteins (CHMPs) that are recruited from monomeric cytosolic pools to form a filamentous structure inside the ILV neck. Complex formation is accompanied by a conformational change in the CHMP proteins that exposes C-terminal motifs that bind to MIT (microtubule-interacting and trafficking) domains in interacting proteins such as VPS4, promoting their endosomal recruitment.^11^, ^12^, ^13^, ^14^ VPS4 functions as a hexameric ring which, using energy from ATP hydrolysis, modifies the ESCRT-III complex filaments by unfolding subunits through its central pore—this subunit removal has been proposed to constrict ESCRT-III filaments and tighten the ILV neck.^1^^,^^4^

ESCRT-III proteins and VPS4 also have roles unrelated to membrane modeling, participating in the dynamic control of mitotic spindle morphology and mitotic spindle checkpoint signaling, as well as in multiple aspects of centrosome biology and primary cilium formation. Cells lacking many different individual ESCRT-III or VPS4 proteins develop aberrant nuclei composed of fragmented or interconnected micronuclei, an increased number of centrosomes, multipolar spindles, and abnormal chromosome alignment during metaphase.^15^ In addition, VPS4 dynamically localizes to centrosomes and regulates centrosome function, position, number, and morphology.^15^^,^^16^ Related to this, loss of VPS4 or overexpression of a dominant-negative ATPase-defective VPS4 mutant has also been linked to reduced primary cilium formation independent of ESCRT-III, via a mechanism proposed to involve disrupted centriolar satellite assembly at the centrosome.^16^

Although mutations affecting the ESCRT-III proteins CHMP1A, CHMP2B, and CHMP4B cause autosomal-recessive pontocerebellar hypoplasia (MIM: 614961), autosomal-dominant amyotrophic lateral sclerosis - frontotemporal dementia (MIM: 614696), and autosomal-recessive cataract (MIM: 605387), respectively, somewhat surprisingly other ESCRT-III-related proteins have not been linked to genetic disease.^17^, ^18^, ^19^ In this study, we have identified and functionally characterized multiple *de novo* heterozygous missense mutations in *VPS4A* (MIM: 609982), which cause a neurodevelopmental disorder characterized by severe hypotonia and developmental delay (DD), intellectual disability (ID), structural brain abnormalities including thin corpus callosum and ponto-cerebellar hypoplasia, extrapyramidal neurological dysfunction, congenital cataracts with visual dysfunction, sensorineural deafness, and hematological abnormalities, providing evidence of an essential function of this ATPase in multiple cellular and developmental processes in humans.

## Material and Methods

### Subjects

**Figure 1:**
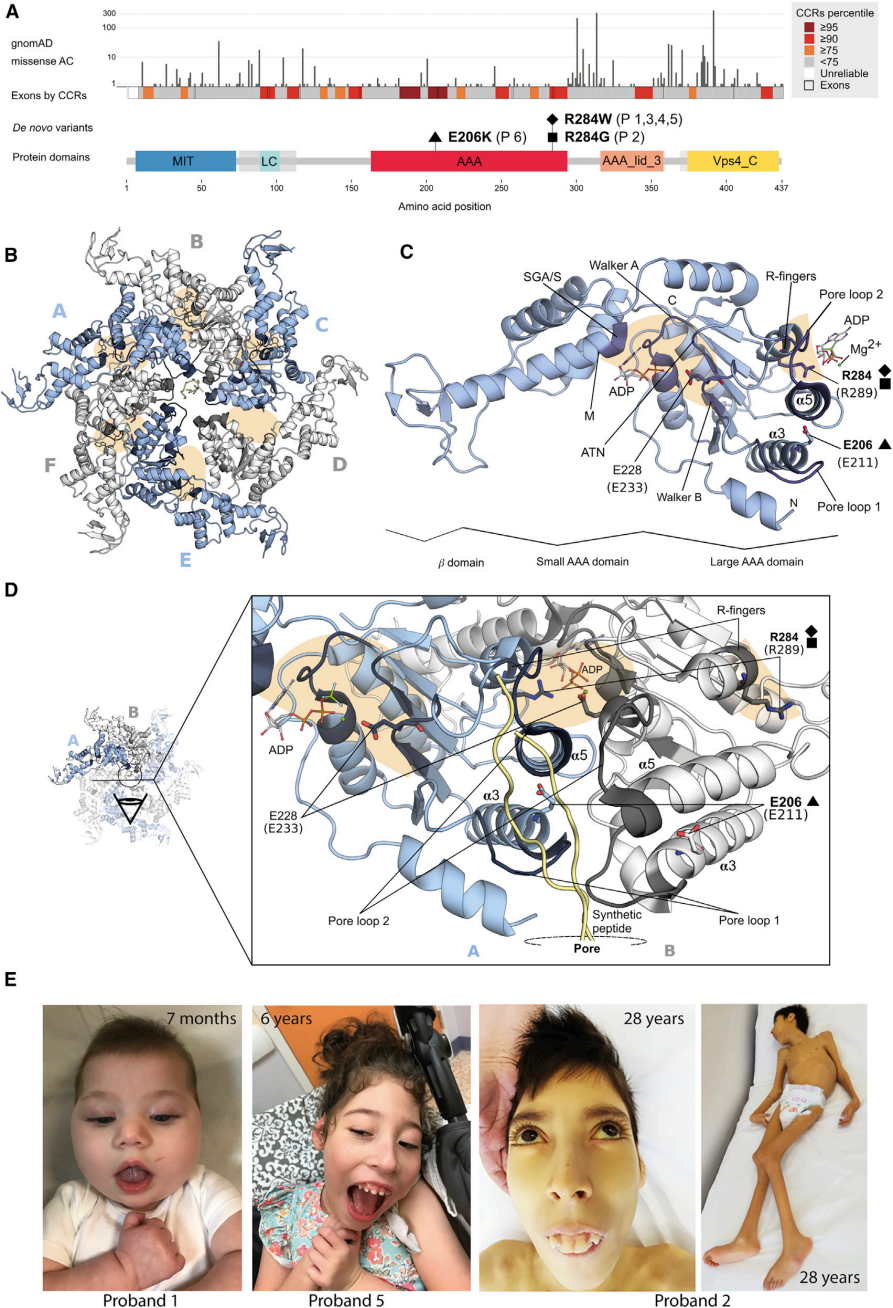
*De Novo* Missense Variants in *VPS4A* Mapped to the Schematic Protein Diagram and the Homologous Yeast Structure (A) Allele count of missense variants in gnomAD and the constrained coding regions’ (CCRs) percentiles are represented for human VPS4A (GeBank: NP_037377) and are aligned with protein domains. The *de novo* missense variants cluster in constrained regions of the large ATPase domain. Coordinates of the protein domains were from Pfam (UniProtKB: Q9UN37). MIT, microtubule interacting and trafficking; AAA, ATPase family associated with various cellular activities; AAA_lid, AAA+ lid domain; Vps4_C, Vps4 C-terminal oligomerization domain; LC, low complexity region. Disordered regions are shaded in translucent gray. (B–D) *De novo* missense variants in VPS4A are mapped to the cryo-EM structure of the ATPase domain of the homologous yeast VPS4 in homohexameric form (PDB: 6OO2). The approximate locations of the active sites are shaded in orange, with the ADP nucleotides represented in sticks, when present. The conserved motifs that define the ATP binding site and pore loops 1 and 2 are shown in dark blue or gray. (B) Structure of the homohexamer, with the six chains alternately colored in blue and white. (C) Structure of a single chain. Both p.Arg284Trp and p.Arg284Gly are observed to affect the R-finger of the active site and p.Glu206Lys affects the intra-chain interface between α3 and α5 helices, located after the pore loops 1 and 2. Only the name of these two helices is shown for clarity. p.Glu228Gln, a rationally designed mutant that produces dominant-negative ATPase-defective VPS4A, falls in the Walker B motif of the active site. (D) Zoom in of the inter-chain interface. The pore loops 1 and 2 shape the pore and interact with the synthetic peptide (in yellow) that shows how the ESCRT-III protein would translocate through the pore. (E) Images of probands at representative ages.

Clinical data and DNA specimens were collected, stored, and used following procedures in accordance with the ethical standards of the declaration of Helsinki protocols, with signed informed consents from the participating subjects or families. The study was approved by the local Institutional Ethical Committee of the Ospedale Pediatrico Bambino Gesù, Rome (1702_OPBG_2018) and the Cambridge South Research Ethics Committee (13/EE/0325). All probands except proband 5 were analyzed in the context of dedicated research projects focused on undiagnosed disorders, while proband 5 was referred for diagnostic genetic testing. Explicit permission was obtained to publish the photographs of the subjects shown in Figure 1.

### Genomic Analyses

WES and WGS was performed using DNA samples obtained from leukocytes. A trio-based strategy was used in all cases. WES and WGS data processing, and variant filtering and prioritization by allele frequency, predicted functional impact, and inheritance models were performed as previously reported.^20^, ^21^, ^22^ The *de novo* origin of the *VPS4A* mutations was confirmed by Sanger sequencing (primer sequences available on request).

### Protein Sequence Conservation and Mapping of Variants to Homologous VPS4 Protein Structures

Sequence conservation of VPS4 proteins was analyzed across orthologous and paralogous protein sequences for different model species as previously described.^23^ Constrained coding regions model was run for gnomAD 2.1.1 exomes.^24^

There are protein structures homologous to human VPS4A in hexameric or monomeric forms in Protein Data Bank (PDB).^25^ Because the hexamer is the functional form of VPS4A, first we mapped all the *de novo* missense variants to the homologous yeast VPS4, since it is the most similar structure to human available in this oligomeric state. This structure has ADP bound and a cyclic peptide in the central pore (PDB: 6OO2, cryo-EM with resolution 4.4 Å).^26^ Then, we compared our observations to the monomeric forms using the homologous human and mouse VPS4B, all X-ray crystal structures. These were in apo forms (human PDB: 1XWI, with resolution 2.8 Å; mouse PDB: 2ZAM, with resolution 3.5 Å) and in ATP- or ADP-bound forms (mouse PDB: 2ZAN and 2ZAO, with resolution 2.8 Å and 3.2 Å, respectively).^27^^,^^28^ All structures were visualized and aligned with PyMOL version 2.0 (The PyMOL Molecular Graphics System, Version 2.0 Schrödinger, LLC).

### Antibodies

Antibidies used: rabbit polyclonal anti-beta III tubulin (ab18207), rabbit polyclonal anti-CHMP2B (ab33174), mouse monoclonal anti-M6PR (ab2733), mouse monoclonal anti-MAP2 (ab11268), rabbit polyclonal anti-pericentrin (ab4448), rabbit polyclonal anti-Tau (ab64193) from Abcam; mouse monoclonal anti-CD63 (clone H5C6; Developmental Studies Hybridoma Bank, University of Iowa); rabbit polyclonal anti-Cathepsin D (219361), rabbit polyclonal anti-Histone H2A.X (07-627), mouse monoclonal anti-myc (clone 4A6, 05-724) from EMD Millipore; mouse monoclonal anti-TfnR (13-6800), mouse monoclonal Anti-BrdU (B35130) from Invitrogen; IRDye-conjugated secondary antibodies from LICOR; rabbit polyclonal anti IST1 (51002-1-AP) from Proteintech Group; mouse monoclonal anti-SNX1 (611582), anti-EEA1 (610456), anti-Rab5 (610742) from BD Transduction Laboratories; mouse monoclonal anti-CHMP6 (clone B-3; sc-398963), mouse monoclonal anti-EGFR (clone A-10, sc-373746), mouse monoclonal anti-LAMP1 (H4A3), mouse monoclonal anti-lamin A/C (E-1: sc-376248), mouse monoclonal anti-acetylated α-tubulin (sc-23950), mouse monoclonal VPS4A (clone A-11, sc-393428) from Santa Cruz Biotechnology; horseradish peroxidase (HRP)-conjugated secondary antibodies, mouse monoclonal anti-β-tubulin (T4026) from Sigma-Aldrich; rabbit polyclonal anti-GAPDH (2118), mouse monoclonal anti-Nanog (clone 1E6C4, 4893), rabbit polyclonal anti-oct-4 (2750), rabbit polyclonal anti-Sox2 (2748) from Cell Signaling Technology; Alexa Fluor 488-, 594-, and 568-labeled secondary antibodies for immunofluorescence from Molecular Probes.

### Constructs

Lentiviral plasmids used the A62 backbone and the packaging plasmids pMD VSV-G and pCMV Δ8.91. A62-myc-*VPS4A* was generated by cloning *VPS4A* from pLNCX2-mCherry-*VPS4A* into the A62 vector (NheI-EcoRI) with the addition of an N-terminal myc tag. Mutant versions of A62-myc-*VPS4A* (p.Arg284Gly, p.Arg284Trp, p.Glu228Gln, p.Glu206Lys, p.Ile337Val, and p.Pro168Ser) were generated by site-directed mutagenesis. sgRNA sequences targeting the transcription start site of *VPS4A* were selected from the Weissman CRISPRi-v2 library.^29^ Sense and antisense sgRNA oligonucleotides were designed with 5′CACC and 3′CAAA overhangs, respectively, and cloned into pKLV-U6gRNA-EF(BbsI)-PGKpuro2ABFP (BpiI) for lentivirus production. The following sgRNA were used: scrambled: GGGACGCGAAAGAAACCAGT; VPS4A G1: GGCAGGGCGGCCGCTCGCAG; VPS4A G2: GACTCGGCTCCCGCTGCGAG; VPS4A G3: GGGAGCCGAGGTACTGGGTC. pLNCX2-mCherry-VPS4A was a gift from Sanford Simon (Addgene plasmid # 115334; RRID: Addgene_115334). pKLV-U6gRNA-EF(BbsI)-PGKpuro2ABFP was a gift from Kosuke Yusa (Addgene plasmid # 50946; RRID: Addgene_50946). A62 was a gift from Michael Fernandopulle (University of Cambridge, UK).

### Cell Culture

Proband fibroblasts, HeLaM, and HEK293T cells were grown in complete Dulbecco’s Modified Eagle’s Medium (DMEM, Sigma-Aldrich) supplemented with 10% fetal bovine serum (FBS, Sigma-Aldrich), 100 U/mL penicillin and 100 μg/mL streptomycin (Sigma-Aldrich), and 2 mM L-Glutamine (Sigma-Aldrich). Human i^3^N and CRISPRi-i^3^N induced pluripotent stem cells (iPSCs;generated in a WTC11 iPSC background line) were a gift from Michael Ward (NIH). iPSCs were cultured in TESR-E8 (STEMCELL Technologies) on dishes coated with Matrigel Matrix (Corning). TESR-E8 was replaced daily and cells were passaged at 80%–90% confluency with 0.5 mM EDTA to maintain colony growth and with the ROCK inhibitor Y-27632 (10 μM, Tocris). CRISPRi-i^3^N iPSCs stably expressing pKLV were additionally cultured in the presence of 2 μg/mL puromycin (Sigma-Aldrich). All cell lines were cultured with 5% CO_2_ at 37°C and were regularly tested for mycoplasma contamination.

### HeLa Cell Transfection

HeLa cells were transfected with purified plasmid using polyethylenimine (PEI, Sigma-Aldrich). In brief, a mixture of 50 μL optiMEM and 1.69 μg of DNA was prepared and incubated at room temperature for 5 min. Another mixture containing 150 μL optiMEM and 3.38 μL PEI was prepared and incubated for 5 min. Both solutions were then mixed together and incubated for 20 min. The total volume was then added to one well of a 6-well plate, already containing 1.5 mL of DMEM supplemented with 10% FBS and 2 mM L-Glutamine. Cells were typically transfected 24 h after plating and incubated with transfection reagents for 29 h.

### I^3^Neuron Differentiation

Differentiation into i^3^Neurons was as previously described, with slight modifications.^30^ Briefly, on day 0 iPSCs were dissociated into single cells using StemPro Accutase (Thermo Fisher Scientific) and seeded at a density of 150,000 cells/cm^2^ on Matrigel-coated culture dishes in Induction Medium (IM) composed of DMEM/F-12, 1× N-2 Supplement, 1× MEM Non-Essential Amino Acids Solution, 1× GlutaMAX Supplement (all Thermo Fisher Scientific), 10 μM Y-27632, and 2 μg/mL doxycycline hydrochloride (Sigma-Aldrich). Pre-differentiated cells were maintained in IM for 3 days with daily medium changes. After the 3-day differentiation period, cells were dissociated with StemPro Accutase and seeded at 5 × 10^4^ cells/cm^2^ onto culture plates coated with 0.1 mg/mL poly-L-ornithine (Sigma-Aldrich). Cells were maintained in Cortical Neuron Culture Medium, composed of BrainPhys Neuronal Medium (STEMCELL Technologies), 1× B-27 Supplement (Thermo Fisher Scientific), 10 ng/mL BDNF (PeproTech), 10 ng/mL NT-3 (PeproTech), and 1 μg/mL mouse Laminin (Thermo Fisher Scientific) with half media changes carried out every 3–4 days.

### Stable Cell Lines

Stable cell lines were generated by lentiviral transduction of iPSCs with the VPS4A and sgRNA lentivectors described earlier. Briefly, HEK293T cells were co-transfected with a lentiviral expression construct and the packaging vectors pCMVΔ8.91 and pMD VSV-G at a ratio of 1:0.7:0.3 using TransIT-293 (Mirus Bio) as per the manufacturer’s instructions. The viral supernatant was collected 48 h post-transfection, passed through a 0.45 μm filter, and added to target cells in the presence of 10 μg/mL polybrene (Sigma-Aldrich). Typically, following spinoculation at 1,800 rpm for 1 h at 32°C, cells were transduced for 16 h. Transduced cells were selected by adding puromycin at a final concentration of 1 μg/mL from 24 h if required.

### Immunoblotting

Cells were washed twice on ice with PBS and subsequently scraped with ice-cold Triton X-100 lysis buffer (1% Triton X-100, 150 mM NaCl, 50 mM HEPES [pH 7.4], 1 mM EDTA, 10% (v/v) glycerol and protease inhibitors). Samples were centrifuged at 20,000 × *g* for 10 min at 4°C. Sample buffer was added to supernatant and samples were heated at 95°C for 5 min. Proteins were resolved by SDS-PAGE and transferred to a PVDF membrane. Membranes were blocked in 5% (w/v) skimmed milk powder in PBS containing 0.1% Tween 20 for 30 min at room temperature before being probed with primary and secondary antibodies. Membranes were visualized using an ECL Western Blotting Detection Kit (GE Healthcare) for HRP-conjugated antibodies or, for IRDye-conjugated secondary antibodies, imaged directly for infrared fluorescence signal detection on an Odyssey Infrared Imaging System using LICOR Image Studio software (LICOR, US). Western blots were quantified by densitometry using ImageJ.

### Analysis of Centrosome and Mitotic Spindle Number, Morphology, and Chromosome Segregation

After 24 h of culture in complete medium, fibroblasts were treated with 2 mM thymidine (Sigma-Aldrich) for 24 h, washed with PBS 1×, recovered with complete medium for 3 h, and then treated with 100 ng/mL nocodazole (Sigma-Aldrich) for 12 h. Afterward, fresh drug-free medium was added and recovery was allowed for the different time points (15 to 120 min) by fixing cells every 15 min using PHEMO buffer for 10 min at room temperature.

### Primary Cilium Analysis

Cells were plated onto coverslips, maintained for 24 h in low serum medium to promote emission of cilia and then fixed in absolute chilled methanol for 10 min at −20°C.

### BrdU Assay

Assessment of cells in the different cell cycle phases was performed by dual flow cytometry analysis of cells incorporating BrdU and stained with the fluorescent DNA probe propidium iodide (PI). Briefly, cells were incubated for 1 h with BrdU (Sigma-Aldrich) at a final concentration of 30 μM. Then, BrdU was removed, cells were rinsed with PBS prior harvesting, and permeabilized using ice-cold 100% ethanol. Cells were incubated with HCl 3N to denature DNA, and 0.1 M sodium tetraborate to stop this reaction. Finally, fibroblasts were incubated with an anti-BrdU antibody followed by goat anti-mouse Alexa Fluor 488 secondary antibody. Cells were then re-suspended in a buffer containing 10 μg/mL RNase A and 20 μg/mL PI and immediately analyzed by FACS.

### Cytokinesis-Block Micronucleus and Chromosome Aberration Assays

The cytokinesis-block micronucleus assay was conducted following a previous protocol.^31^ In actively dividing cells, cytokinesis was blocked with 4.5 μg/mL cytochalasin B (Sigma-Aldrich), an inhibitor of actin polymerization. Twenty-four hours later, cells were collected by cytospin centrifugation (Shandon Cytospin 3, Thermo Fisher Scientific) at 600 r.p.m. for 5 min and fixed in absolute methanol at −20°C for 10 min. Slides were stained with 3% Giemsa (Sigma-Aldrich) in Sorensen buffer (pH 6.8), and the analysis was performed by using an optical microscope. Cells were analyzed following previously reported criteria.^31^

Chromosome aberrations were analyzed in mitotic cells obtained from actively dividing cells treated for 2 h with colcemid (0.1 μg/mL, Sigma-Aldrich). Cells were harvested by standard procedures. Briefly, after 10 min incubation at 37°C in 0.075 M KCl, fibroblasts were fixed three times with cold methanol/acetic acid (3:1). Slides were prepared by a conventional air-drying technique and stained with 5% Giemsa in Sorensen buffer (pH 6.8).

### Immunofluorescence Microscopy on Fixed Cells

Cells were fixed at room temperature in 3.7% (v/v) formaldehyde in PBS and permeabilized in PBS containing 0.1% (v/v) saponin (Sigma-Aldrich) or 0.1% (v/v) Triton X-100 (Sigma-Aldrich). Coverslips were labeled with primary and secondary antibodies as previously described.^32^ Slides were analyzed with a LSM980 confocal microscope (63× NA 1.40 oil immersion objective, 37°C), LSM880 confocal microscope (100× or 63× NA 1.40 oil immersion objective, 37°C), LSM780 confocal microscope (63× NA 1.40 oil immersion objective, 37°C), Leica TCS SP2 AOBS confocal microscope (63× NA 1.40 oil immersion objective, 37°C), or an AxioImager Z2 Motorized Upright Microscope (100× or 63× NA 1.40 oil immersion objective, room temperature, Axiocam 506; ZEISS). Images were subsequently processed using Huygens Professional software for deconvolution, ImageJ, Adobe Photoshop, and Adobe Illustrator.

### Image Analysis and Quantitation

To determine the percentage of cells with large marker-positive endosomes, the largest organelle per cell was measured using ImageJ, with at least 100 cells recorded per experimental condition. For quantification of the mean number of puncta per cell, images of ≥20 cells per condition were analyzed using the ImageJ “analyze particles” command. Co-localization analysis was performed in one of two methods. (1) Individual puncta were first delineated by intensity thresholding in ImageJ and the number of co-localized puncta were then counted manually for 5 cells (proband fibroblasts) or ≥20 cells per condition per experimental replicate (i^3^Neurons). (2) Alternatively, the extent of co-localization was determined by calculating the Pearson’s correlation coefficient for red and green pixels in each cell using the Coloc2 ImageJ plugin for 10 cells per condition. To assess the percentage of cells with aberrant centrosome number, ≥25 cells were analyzed in each of the 6 biological repeats for each experimental condition (200 cells/line). To assess the percentage of cells with aberrantly shaped nucleus, ≥40 cells were analyzed in each of the 4 biological repeats for each experimental condition (200 cells/line). To assess the percentage of cells with micronuclei and nucleoplasmic bridges, 200 cells (micronuclei) or 250 (nucleoplasmic bridges) were analyzed in each of the 5 (micronuclei) or 4 (nucleoplasmic bridges) biological repeats for each experimental condition (1,000 cells/line). For the analysis of chromosome aberrations, ≥30 well-spread metaphases were analyzed in each of the 3 biological repeats for each experimental condition (100 cells/line). Since fixation procedures may often result chromosome loss, the analysis was restricted to metaphases containing 45–46 chromosomes. To assess DNA damage, ≥40 cells were analyzed in each of the 4 biological repeats for each experimental condition (200 cells/line), counting only cells showing more than 20 foci positive to γ-H2AX staining. Finally, for determining the amount of cells with aberrant primary cilium, a total of 100 cells were analyzed for each cell line over two experiments.

### Statistical Analysis

Statistical analysis and post hoc tests were carried out as described in figure legends using GraphPad Prism 8. The statistical significance is denoted on graphs by asterisks (^∗^), where ^∗^p < 0.05, ^∗∗^p < 0.01, ^∗∗∗^p < 0.001, and n.s. = not significant.

## Results

### Genetic Analysis

Using data from GeneMatcher, the UK National Institute for Health Research Bioresource and Genomics England Research Consortium,^33^ or repositories linked to diagnostic testing (GeneDx Laboratory), six unrelated individuals with *de novo* variants in *VPS4A* (GenBank: NM_013245.3) were identified. All had a common and distinctive phenotype including microcephaly, profound neonatal onset of hypotonia, and global developmental delay, with similar structural brain abnormalities and cataracts in the majority.

Five of the probands had *de novo* heterozygous missense variants at amino acid position 284. These included four (probands 1, 3, 4, and 5) who had a c.850A>T (p.Arg284Trp) substitution and a single case (proband 2) with a c.850A>G (p.Arg284Gly) change. Three of these case subjects were identified from a trio-based whole-genome sequencing (WGS) approach (probands 1, 3, and 4),^22^^,^^34^ while the others were identified using trio-based whole-exome sequencing (WES) in the context of the Undiagnosed Patients Program at the Ospedale Pediatrico Bambino Gesù (proband 2) or within routine care by GeneDx Laboratory (proband 5). In addition, trio WGS in a further case identified a *de novo* variant at c.616G>A (p.Glu206Lys; proband 6). In each case, there was a single plausible *de novo* variant in the absence of any pathogenic variant in genes previously associated with Mendelian diseases. Subsequent WGS in proband 2 further excluded the occurrence of other clinically relevant variants. The DNA variants causing the p.Arg284 and p.Glu206 alterations were not present in control population databases (gnomAD, ExAC, TOPMed), fell in regions highly constrained for variation in control populations (Figure 1A), and affected conserved amino acids of the AAA ATPase domain of VPS4A that are invariable across VPS4 family members from multiple species (Figure S1). They had strong computational evidence for pathogenicity (Table S1).

Two DNA variants—c.502C>T (p.Pro168Ser) and c.1009A>G (p.Ile337Val)—were also observed in one and three unrelated families, respectively, from the Genomics England Research Consortium. These were also absent from gnomAD. However, the mode of inheritance of the variants was unavailable and the pathogenicity of these substitutions was less plausible computationally, with lower CADD (25 and 23) and REVEL scores (0.911 and 0.336), respectively. Additionally, the clinical phenotype of the probands was of non-specific ID, without the distinctive features that were prominent in the other subjects, so these variants were assessed as having uncertain significance.

### Mapping of Putative Pathogenic Missense Variants in VPS4A to Homologous VPS4 Protein Structure

Protein structural mapping of the putative pathogenic variants to the yeast VPS4 homologous protein structure revealed their proximity to the ATP catalytic site and the pore lining loops. Arg284 was a hot-spot of *de novo* mutations in our subjects. It is one of two arginine residues known as the arginine fingers, a motif that is part of the interface between chains of the hexamerized VPS4, where the catalytic active site is assembled (Figures 1B–1D). Mutations of equivalent arginine finger residues have been studied in multiple ATPases and cause complete loss of *in vitro* or *in vivo* catalytic activity of the protein, indicating that they are necessary for ATP hydrolysis.^35^ The arginine fingers may also be important for oligomerization of ATPase protein complexes.^35^ Thus, existing functional data strongly support a deleterious effect of mutations affecting Arg284.

In contrast, the Glu206 residue is functionally uncharacterized. It is located by the N-terminal of the α3 helix in the boundary with the pore loop 1 (Figures 1B–1D). The lateral chain of Glu206 (Glu211 in yeast) points to the α5 helix that is on top and connected to pore loop 2 in all the chains of the hexamer (Figures 1B–1D and S2A). Pore loops 1 and 2 are very flexible and shape the central pore of the hexamer in ATPases. In the yeast structure both interact with a synthetic peptide that mimics ESCRT-III proteins, highlighting their role on the translocation of these proteins through the pore (Figure 1D).^26^ The replacement of Glu206 by lysine introduces a change from a negative to a positively charged and slightly bigger lateral chain. Additionally, mapping of this position to the monomeric human and mouse VPS4B shows that the Glu213 (equivalent to Glu206) adopts different orientations, and the N terminus of the α3 helix is slightly unfolded with the coordinates of pore loop 1 unresolved (Figure S2B), suggesting a possible conformational change in this region between the monomeric and hexameric forms. Therefore, we suggest that this variant is likely to affect the fold of the N terminus of α3 helix, the flexibility of the pore 1 loop, and the lateral interactions between α3 and α5 helices, hence resulting in alterations in the recognition and translocation of ESCRT-III proteins.

### Clinical Profile of Probands

The six probands with *de novo* substitutions affecting Glu206 or Arg284 had a consistent phenotype characterized by severe DD, profound ID, and dystonia (Figures 1E and S3, Table S1, see Supplemental Note). Children were very delayed in establishing head control and none achieved independent walking. Other common findings were cerebellar hypoplasia (five individuals out of six, the other showing uncharacterized severe cerebral atrophy) with a variable degree of corpus callosum hypoplasia. One individual also had bilateral polymicrogyria. Epilepsy was present in three and dystonia in five subjects. Eye involvement was also a common finding, including congenital cataract, retinal dystrophy, and in one case congenital Leber amaurosis. Four individuals were diagnosed with hepatosplenomegaly and/or steatosis. Three subjects had anemia, which was characterized as dyserythropoietic in two. Severe feeding difficulties were present in four individuals, requiring assisted feeding in three. Two had sensorineural deafness. Severe growth retardation, generally for all parameters, was present in most cases. Notably, severe microcephaly (typically with Z scores < −5) was universal. Overall, the disorder seems to have a poor prognosis as two affected individuals died in childhood or early adult life (Table S1).

In general, the affected individuals presented with a complex and severe phenotype with some features reminiscent of a ciliopathy-related disorder (cerebellar hypoplasia, retinal dystrophy or Leber amaurosis, sensorineural deafness), a significant neurodevelopmental condition (severe microcephaly and ID), and other features including cataracts, hepatosplenomegaly, and congenital anemia, giving rise to a distinct syndrome.

### VPS4A Disease-Associated Variants Have a Dominant-Negative Effect on Endosomal Morphology

**Figure 2:**
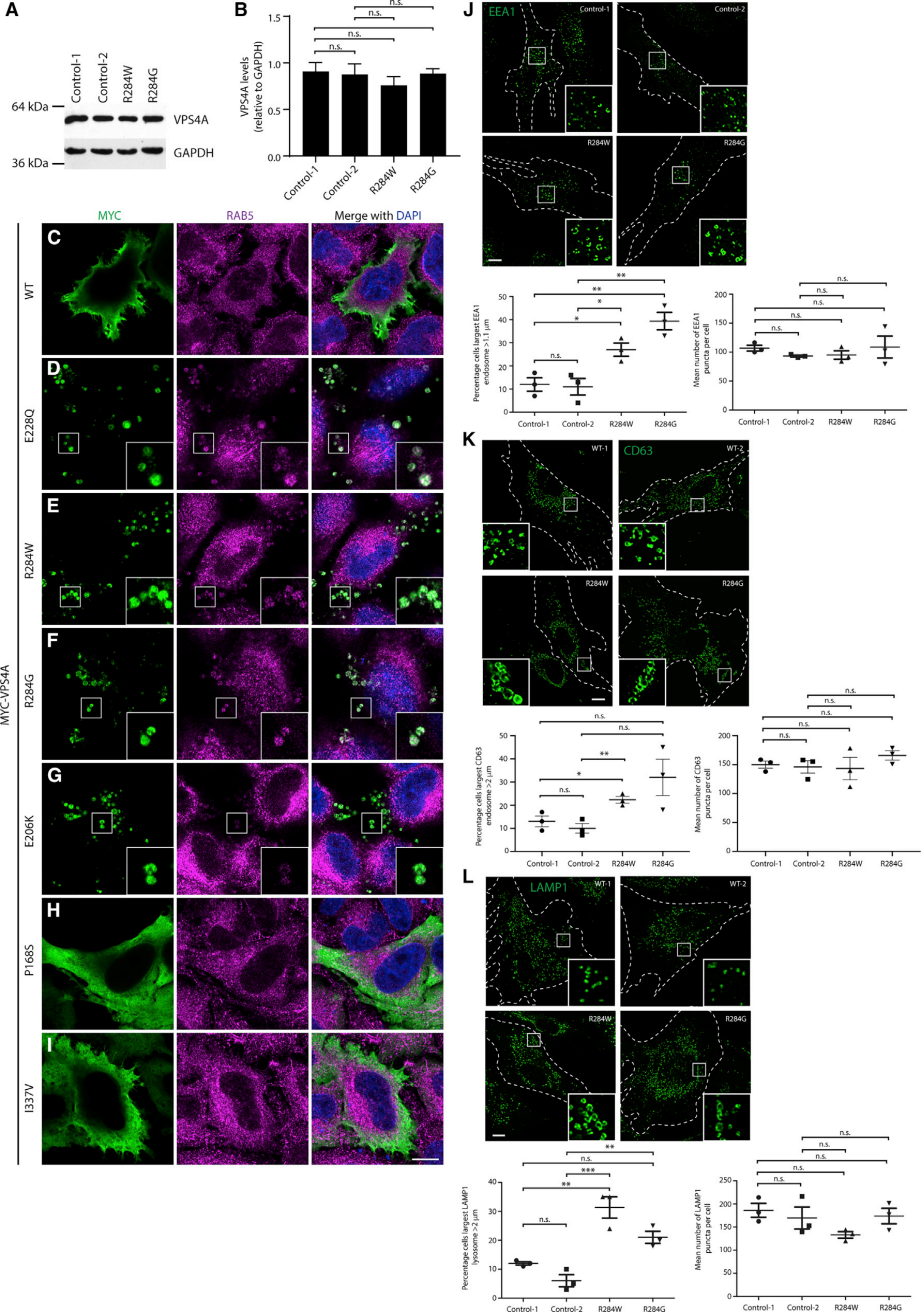
VPS4A Disease-Associated Variants Have a Dominant-Negative Effect on Endosomal Morphology (A) Representative VPS4A immunoblots of fibroblast cell lysates from a proband (proband 1) with the p.Arg284Trp sequence change and her parents (control 1 and control 2), and from a proband (proband 2) with the p.Arg284Gly sequence change. (B) Immunoblot band intensities from three such experiments were quantified, normalized to GAPDH loading control values, and plotted in the corresponding graph. (C–I) HeLa cells were transfected with constructs expressing wild-type myc-VPS4A, myc-VPS4A containing the rationally designed ATPase-defective p.Glu228Gln mutant, or myc-VPS4A harboring the sequence changes identified in probands. Cells were fixed, labeled with anti-Myc and anti-RAB5 antibodies, and visualized with confocal microscopy. The inset panels show higher magnification views of the boxed regions; examples of large vacuolar endosomal structures are shown. (J–L) Cultured fibroblasts from the control subjects and probands indicated were fixed, labeled with EEA1 (early endosomes) (J), CD63 (preferentially labels late endosomes) (K) and LAMP1 (predominantly labels lysosomes) (L), then visualized by widefield immunofluorescence microscopy. The percentage of cells with an endosomal organelle over a nominal cut-off size and the number of labeled organelles per cell was counted in n = 3 biological repeats for each marker (in 100 cells per experimental condition in each repeat), then quantified in the corresponding charts. Bars in all plots show mean ± SEM, p values calculated by one-way ANOVA with Tukey’s post hoc test for repeated-measures. Scale bars = 10 μm.

To investigate the molecular pathological mechanism of the disease-associated VPS4A alterations, we first examined the cellular expression of VPS4A and found no alteration in protein abundance in fibroblasts from individuals with *de novo* p.Arg284Trp or p.Arg284Gly substitutions versus healthy parental control subjects, suggesting that mutant protein stability is unaltered (Figures 2A and 2B).

The published, rationally designed VPS4A-p.Glu228Gln mutant and the equivalent VPS4B mutant (p.Glu235Gln) are ATP hydrolysis defective. Cultured cells overexpressing either of these mutants develop significantly enlarged endosomal vacuoles, caused by a dominant-negative effect of incorporation of the ATPase-defective protein into VPS4 hexamers and subsequent failure of disassembly of the ESCRT complexes, which in turn makes ESCRT proteins unavailable for subsequent rounds of ILV formation.^36^, ^37^, ^38^

We used this phenotype to interrogate whether the identified disease-associated variants have a dominant-negative effect on VPS4A function. We first confirmed that, as expected, expression of VPS4A-p.Glu228Gln in HeLa cells caused an enlarged endosomal vacuolar phenotype, in contrast to wild-type VPS4A, which exhibited a pan-cytosolic distribution consistent with its known localization pattern (Figures 2C and 2D). Expression of VPS4A-p.Arg284Trp, VPS4A-p.Arg284Gly, or VPS4A-p.Glu206Lys caused the development of vacuolar endosomal structures identical to those generated by VPS4A-p.Glu228Gln expression (Figures 2E–2G). In contrast, the localization pattern of VPS4A-p.Pro168Ser and VPS4A-p.Ile337Val resembled that of wild-type VPS4A (Figures 2H and 2I). We concluded that the p.Arg284Trp, p.Arg284Gly, and p.Glu206Lys amino acid substitutions exert a dominant-negative effect on VPS4 hexamer function, while the p.Pro168Ser and p.Ile337Val sequence changes do not; considered with the clinical and bioinformatics data above, this suggests that the latter two variants are unlikely to be pathogenic.

### Physiological Expression of Disease-Associated VPS4A Causes Abnormal Endolysosomal Morphology in Proband Fibroblasts

We examined whether physiological expression of heterozygous p.Arg284Trp or p.Arg284Gly mutants in proband-derived fibroblasts caused altered endosomal morphology. We did not observe extremely large vacuolar structures of the type observed in cells overexpressing exogenous ATPase-defective VPS4. However, the proband-derived cell lines had an increase in the percentage of cells with larger vesicles labeled by EEA1 (an early endosome marker), CD63 (which is typically enriched in the ILVs of the late endosome), and LAMP1 (enriched in lysosomes), in the absence of significant alterations in the total number of endosomal structures of each compartment (Figures 2J–2L). We did not observe any increased co-localization between early endosomal, late endosomal, or lysosomal markers in proband cells (Figure S4). Thus, heterozygous expression of mutant VPS4A at endogenous levels causes significant enlargement of multiple endosomal compartments, without apparent content mixing between them.

### Localization and Known Functions of the Core ESCRT-III Complex at Endosomes Are Not Defective in Proband Fibroblasts

As cells overexpressing dominant-negative ATPase-defective VPS4 develop accumulations of ESCRT proteins on enlarged endosomal structures, we investigated whether the proband fibroblasts showed similar aberrant endosomal ESCRT-III localization. We examined the ESCRT-III protein CHMP2B, a core ESCRT-III component that is recruited to the endosomal membrane during ILV formation. Surprisingly, there was no alteration in the number of cellular CHMP2B puncta with an area greater than 0.1 μm^2^, or in the percentage of EEA1- or CD63-positive endosomes that were associated with CHMP2B puncta (Figures S5A and S5B).

We examined late endosomal ultrastructure in the proband cell lines. Consistent with the light microscopy findings, large endosomes appeared to be more prominent. However, endosomes were still competent to make ILVs, a key function of ESCRT-III, as ILVs within MVBs were readily observed in the proband cells (Figure S5C).

Efficient degradation of the epidermal growth factor receptor (EGFR) involves sorting to the ILVs of the MVB, via a mechanism that requires core ESCRT-III components.^39^ EGFR degradation is inhibited by VPS4B depletion, either alone or combined with VPS4A depletion, or by expression of dominant-negative ATPase-defective VPS4B-p.Glu235Gln.^37^^,^^40^, ^41^, ^42^ To our knowledge, the specific role of VPS4A in this process has not been investigated. We examined EGFR degradation in proband fibroblasts carrying the VPS4A-p.Arg284Trp or VPS4A-p.Arg284Gly mutants and, consistent with the retained competence of these cells to make ILVs and regulate endosomal localization of core ESCRT-III components, we found that it was not inhibited; indeed, EGFR degradation was increased at 180 min post internalization (Figure S5D).

We concluded that heterozygous expression of mutant VPS4A in proband cells does not affect the cellular distribution of a core ESCRT-III complex member, prevent formation of ILVs, or adversely affect the degradation of EGFR.

### The Atypical ESCRT-III Protein IST1 Accumulates on Endosomes in Proband Fibroblasts

**Figure 3:**
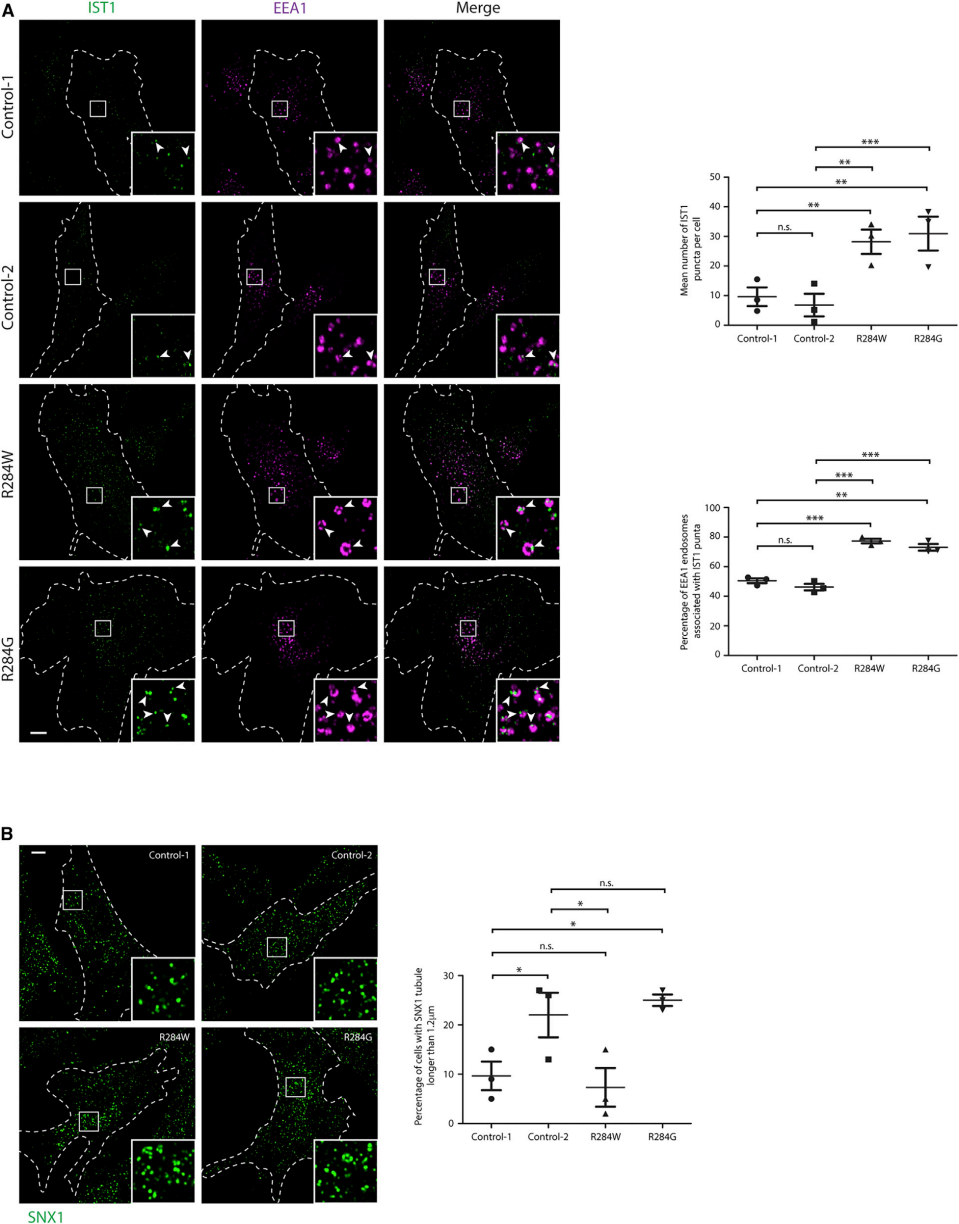
The Atypical ESCRT-III Protein IST1 Accumulates on Endosomes in Proband Fibroblasts (A) Cultured fibroblasts from control subjects or the probands indicated were fixed, labeled against EEA1 and IST1, then visualized with confocal immunofluorescence microscopy. The number of IST1 puncta per cell and the percentage of EEA1-positive endosomes associated with an IST1 punctum was quantified in three such experiments (in five cells per experimental condition in each biological repeat) and plotted in the corresponding charts. Arrows indicate juxtaposed or co-localized puncta. (B) Cultured fibroblasts from control subjects or the probands indicated were fixed and labeled for the endosomal tubular marker SNX1, then visualized with widefield immunofluorescence microscopy. The percentage of cells with at least one SNX-1-positive tubular structure > 1.2 μm in length was quantified in 100 cells per sample, and the results for three such experiments were plotted in the corresponding chart. Bars indicate mean ± SEM, p values calculated by one-way ANOVA with Tukey’s post hoc test for repeated-measures. Micrograph scale bars = 10 μm.

ESCRT-III proteins also play a role in fission of endosomal sorting tubules from the parent endosome. Rather than involving the core ESCRT-III complex, this activity is mediated by a complex formed of two atypical ESCRT-III proteins, IST1 and CHMP1B.^5^^,^^6^^,^^43^ Suitable antibodies are available to visualize IST1 by immunofluorescence. At steady state, IST1 is localized in juxtaposition with early endosomal markers, and it is recruited to endosomal membranes by dominant-negative VPS4A-p.Glu228Gln.^44^ We examined the appearance and localization of IST1 and observed an increased number of IST1 puncta and increased percentage of EEA1-positive endosomes associated with IST1 puncta in proband-derived fibroblasts with the p.Arg284Trp or p.Arg284Gly substitutions, consistent with the idea that VPS4A ATPase activity is required to regulate the association of IST1 with endosomes (Figure 3A). No increased recruitment of IST1 to late endosomes or lysosomes was observed (Figure S6). HeLa cells lacking IST1 show increased tubulation of the endosomal tubular marker SNX1 caused by a failure of endosomal tubule fission,^6^ but we did not consistently observe this phenotype in the proband fibroblasts (Figure 3B).

We concluded that heterozygous *VPS4A* mutations cause aberrant accumulations of the atypical ESCRT-III protein IST1 on endosomal membranes.

### Human Neurons Lacking VPS4A Exhibit Similar Endosomal Phenotypes to Proband Fibroblasts

In view of the prominent neurodevelopmental phenotype observed in probands affected by VPS4A mutants, we attempted to model their effect in human neurons. We first generated i^3^ iPSC lines expressing the VPS4A-p.Arg284Trp or -p.Arg284Gly mutants, using lentiviral transduction of appropriate constructs. In contrast to lines expressing wild-type VPS4A, these lines showed no detectable exogenous VPS4A by immunoblotting, and only very sparse expression (<1% of cells) was observed by immunofluorescence. Those cells that did express the mutant VPS4A exhibited enlarged, vacuolar endosomal structures typical of dominant-negative VPS4 mutants (Figure S7).^36^, ^37^, ^38^ In i^3^ iPSCs, the neurogenic transcription factor NGN2 is integrated under a doxycycline-responsive promoter at a safe harbor locus in the WTC11 iPSC line.^45^ This experimental system allows simple and rapid generation of glutamatergic cortical neurons (i^3^Ns) upon brief culture of the iPSCs in the presence of doxycycline; the cells have morphological and biochemical properties of neurons 14 days post-induction, and are electrically active after 21 days (Figure S8).^45^ However, no VPS4A mutant expressing cells were identified upon differentiation of the i^3^ iPSCs to neurons and we concluded that overexpression of these altered proteins is incompatible with neuronal survival.

**Figure 4:**
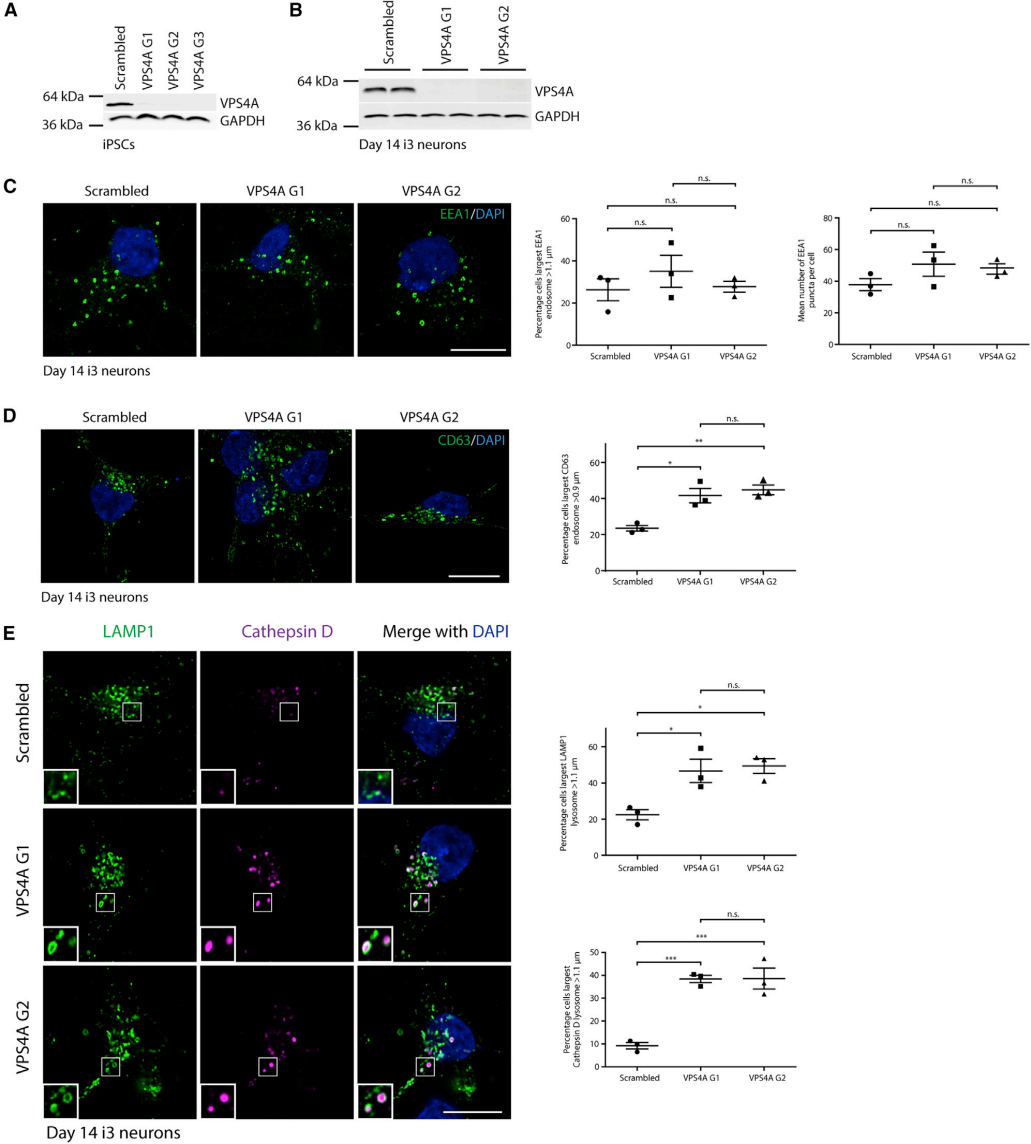
Human Neurons Lacking VPS4A Exhibit Similar Endosomal Phenotypes to Proband Fibroblasts (A) CRISPRi-i^3^N iPSCs with were transduced with a scrambled sgRNA and three separate sgRNAs (G1-G3) directed against VPS4A. Cell lysates were immunoblotted against VPS4A. (B) Selected lines were treated with doxycycline to induce neuronal differentiation, then blotted against VPS4A 14 days later. GAPDH signal validates equal lane loading. (C) CRISPRi-i^3^N iPSCs expressing the guides indicated were differentiated to neurons for 14 days, fixed, and labeled for EEA1. The percentage of cells with an EEA1-positive organelle over a nominal cut-off size was visualized by widefield microscopy and quantified in 3 experiments (≥100 cells per experimental condition in each repeat). The number of EEA1-positive endosomes per cell was visualized using confocal microscopy and quantified in 3 experiments (≥20 cells per experimental condition in each repeat). Quantifications are plotted in the corresponding graphs. (D) i^3^Neurons expressing the sgRNAs indicated were fixed and labeled for CD63, then visualized by widefield microscopy. The percentage of cells with a CD63 organelle over a nominal cut-off size was quantified as described for EEA1. (E) i^3^Neurons expressing the sgRNAs indicated were fixed and labeled for LAMP1 and cathepsin D, then visualized by widefield microscopy. The percentage of cells that had a LAMP1 or cathepsin D organelle over a nominal cut-off size was quantified as described for EEA1. Bars show mean ± SEM, p values generated by one-way ANOVA with Tukey’s correction for multiple testing. Scale bars = 10 μm.

**Figure 5:**
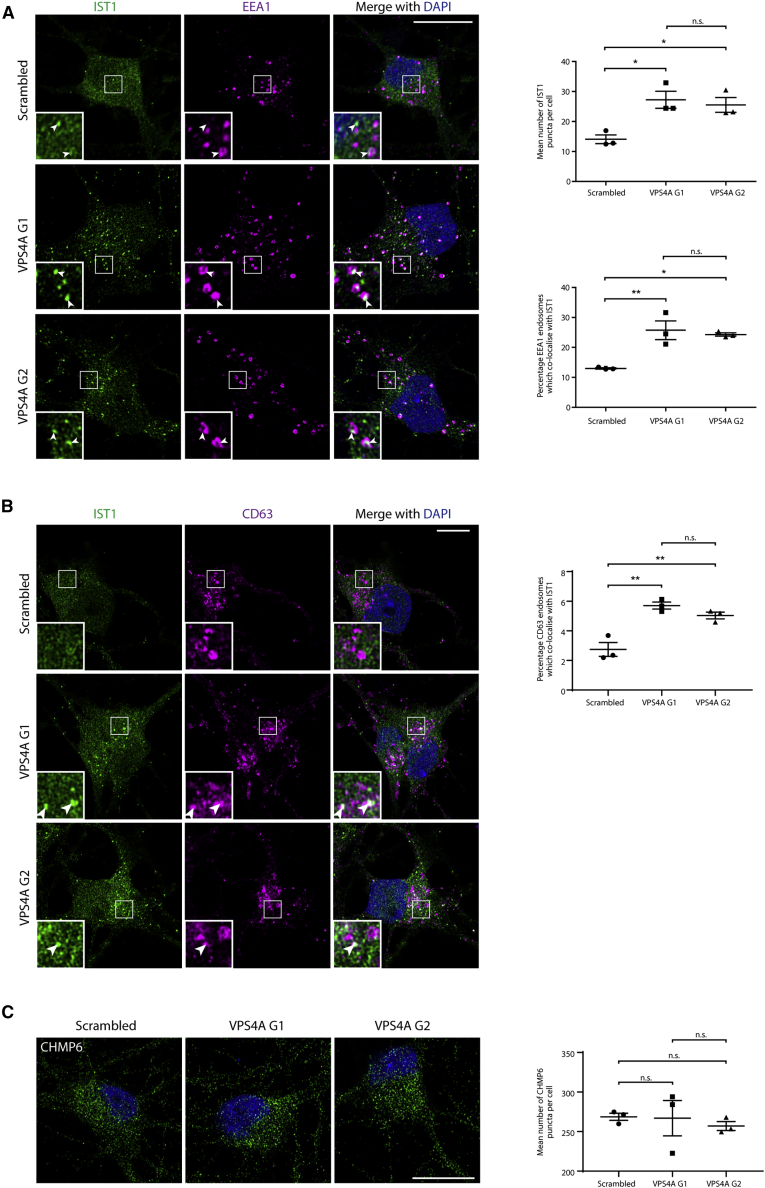
The Atypical ESCRT-III Protein IST1 Accumulates on Endosomes in Human Neurons Lacking VPS4A (A and B) i^3^Neurons expressing the guides indicated were fixed and labeled for IST1 and EEA1 (A) or IST1 and CD63 (B), then visualized by confocal immunofluorescence microscopy. The number of IST1 puncta per cell and the percentage of EEA1- or CD63-positive endosomes associated with an IST1 punctum was quantified in 3 experiments per marker (in ≥20 cells per experimental condition in each repeat) and plotted in the corresponding charts. Arrows indicate juxtaposed or co-localized puncta. (C) i^3^Neurons expressing the guides indicated were fixed and labeled for CHMP6, then visualized by confocal immunofluorescence microscopy. The number of CHMP6 puncta per cell was quantified in 3 biological repeats (≥20 cells per experimental condition in each repeat). Bars indicate mean ± SEM, p values calculated by one-way ANOVA with Tukey’s correction for multiple testing. Micrograph scale bar = 10 μm.

In light of the apparent dominant-negative effect of the overexpressed VPS4A mutants, we reasoned that lack of VPS4A may have similar cellular consequences to heterozygous physiological expression of VPS4A mutants that are capable of blocking the function of the wild-type protein, and so may provide useful insights into the potential functional effects of the mutants in neurons. In addition, analysis of neurons lacking VPS4A will elucidate the physiological role of VPS4A in these cells. We therefore employed a modified i^3^ iPSC system, in which CRISPR-inhibition (CRISPRi) machinery is integrated into a safe harbor locus.^46^ In CRISPRi, an enzymatically dead Cas9 fused to a KRAB transcriptional repressor is targeted close to the transcriptional start site of a target gene by a single guide RNA (sgRNA), thereby inhibiting expression of the gene. This system has advantages over standard CRISPR-based knock-out systems, including high specificity with strikingly few off-target effects and low toxicity.^47^ We targeted *VPS4A* for repression in CRISPRi-i^3^ iPSCs, using two independent sgRNAs, confirmed cellular depletion of VPS4A in the iPSCs, then differentiated each line to i^3^Ns (Figures 4A and 4B). We examined endosomal morphology in these neurons at 14 days differentiation. While there was no significant increase in the percentage of neurons that had enlarged EEA1-positive puncta (Figure 4C), we observed a significant increase in the percentage of cells with enlarged structures marked by CD63, LAMP1, or the lysosomal enzyme cathepsin D (Figures 4D and 4E). In addition, there was an increase in the number of IST1 puncta per cell and of IST1 localization on early and late endosomal structures (Figures 5A and 5B). No difference in the number of puncta of the core ESCRT-III component CHMP6 was observed (Figure 5C).

We concluded that VPS4A regulates endosomal size and endosomal membrane localization of the atypical ESCRT-III protein in human neurons and that loss of VPS4A in neurons largely recapitulates phenotypes that are observed in proband cells expressing dominant-negative VPS4A at physiological heterozygous levels.

### VPS4A Mutants Affect Centrosome and Mitotic Spindle Organization and Are Associated with Aberrant Chromosomal Segregation and G2/M Cell Cycle Arrest

**Figure 6:**
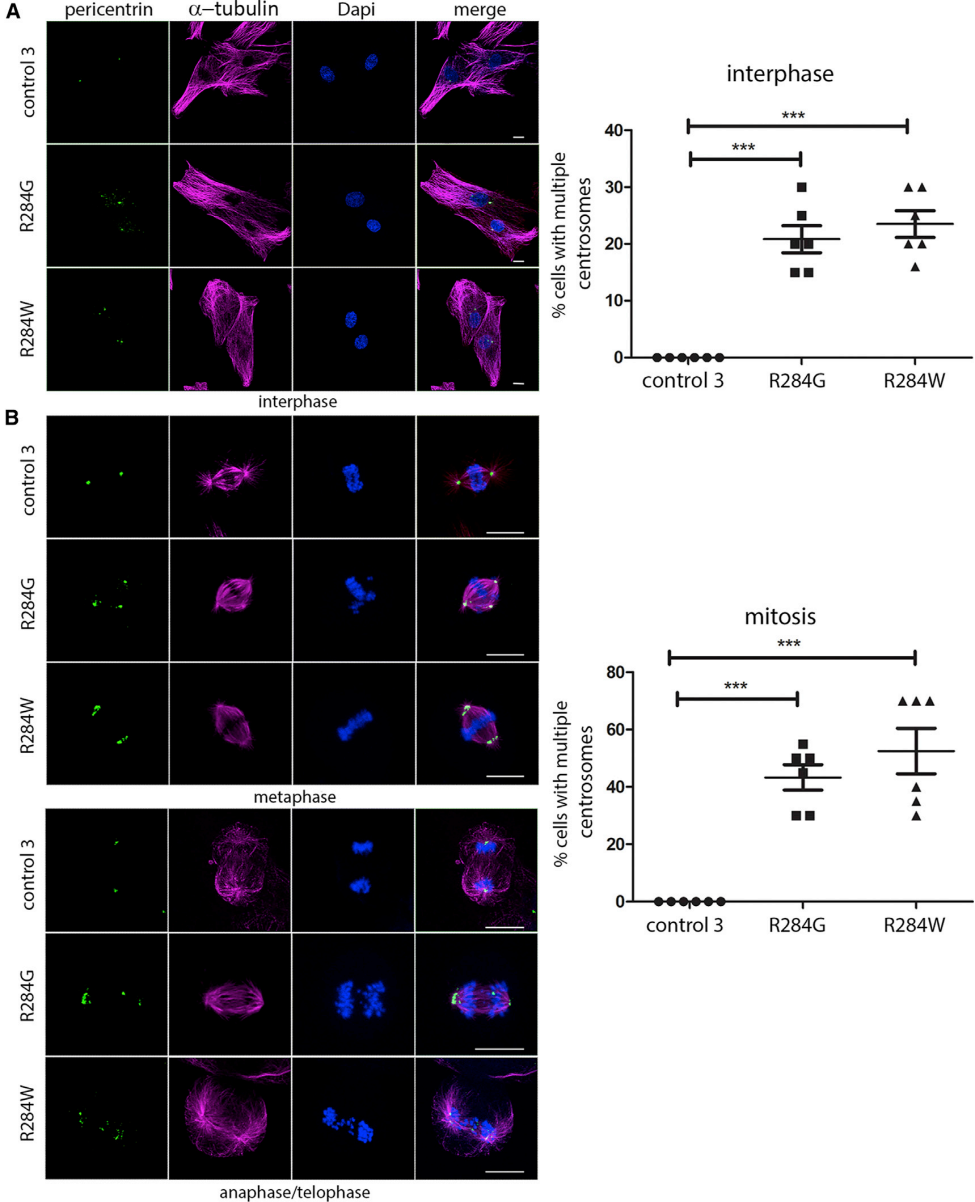
Defective VPS4A Function Affects Centrosome Numbers and Mitotic Spindle Organization Confocal microscopy analysis was performed in synchronized skin fibroblasts from subjects with *VPS4A* mutations and control cells. Images are representative of three stages of the cell cycle (A), interphase (B), metaphase, and anaphase-telophase. Cells were stained using antibodies against pericentrin (centrosome marker) and α-tubulin (microtubules and mitotic spindle); chromosomes with DAPI. Scale bars represent 15 μm. The corresponding graphs show the mean ± SEM of 6 separate counts (≥25 cells/line each) for a total of 200 cells/line scored. p values were calculated by one-way ANOVA with Tukey’s correction for multiple testing.

**Figure 7:**
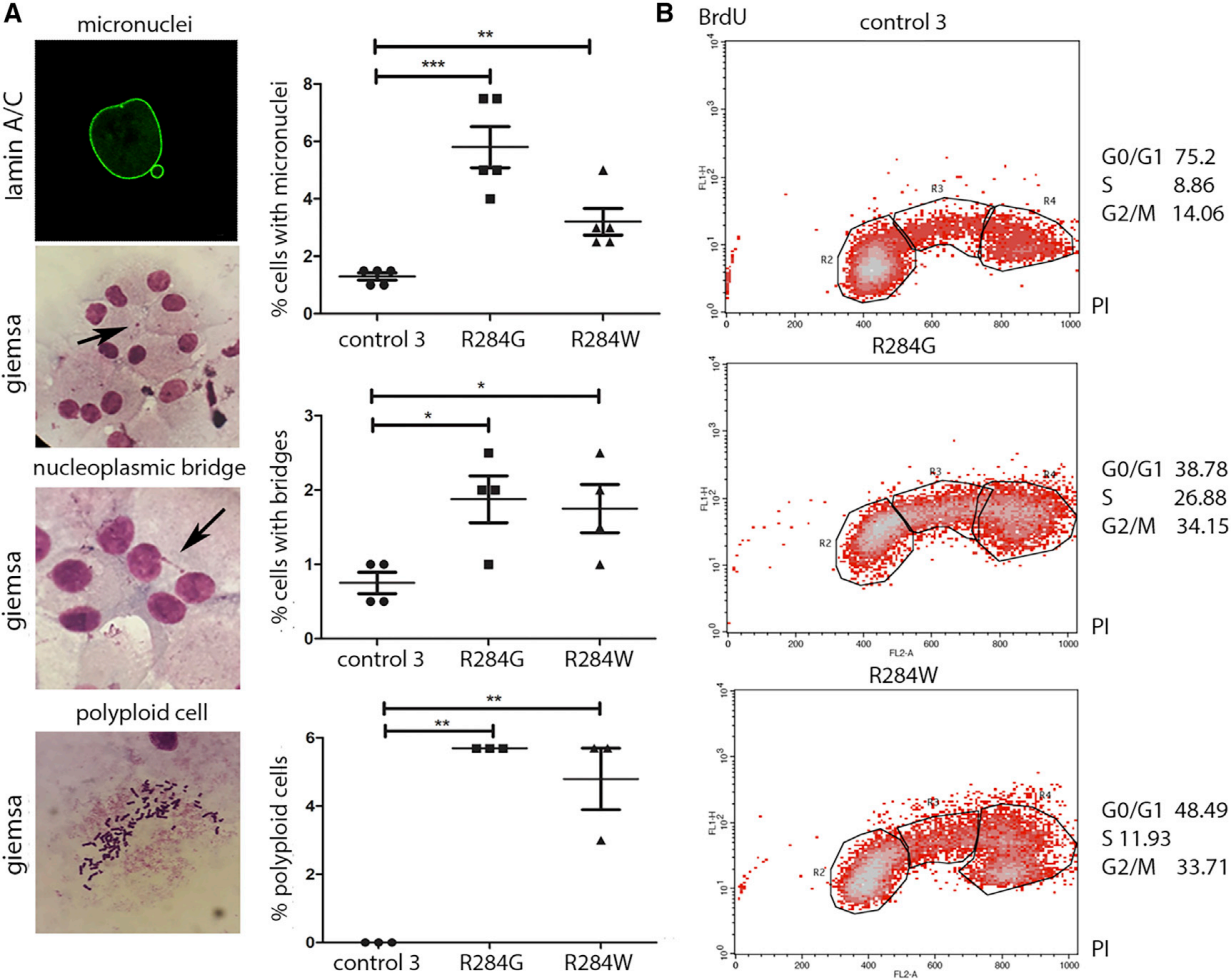
*VPS4A* Mutations Cause Aberrant Chromosome Segregation and Alter Cell Cycle Progression (A) Staining performed using a fluorescent probe (anti-lamin A/C green) or Giemsa show a significant increase in micronuclei (arrow), chromosome bridges (arrow) and aneuploidy in proband cells compared to control cells. In experiments to assess micronuclei and chromosome bridges, graphs show the mean ± SEM of 5 (micronuclei) or 4 (chromosome bridges) separate counts (200 cells/line each, micronuclei; 250 cells/line each, chromosome bridges) for a total of 1,000 cells/line scored. In experiments to assess aneuploidy, graphs show mean ± SEM of 3 separate counts (≥30 cells/line each) for a total of 100 cells/line scored. (B) Cell cycle phases of control subjects’ (top) and probands’ (bottom) fibroblasts as measured by BrdU incorporation and propidium iodide (PI) flow cytometry analysis. The upper box identifies cells incorporating BrdU (S phase), the lower left box identifies G0/G1 cells, and the lower right box represents G2/M cells. A representative of three independent experiments is shown. In all experiments, p values were calculated by one-way ANOVA with Tukey’s correction for multiple testing. Graph bars show mean ± SEM.

Centrosomes serve as solid-state signaling platforms to dynamically regulate a wide array of cellular structures and processes. The ESCRT-III complex and VPS4 proteins are required to maintain normal centrosome morphology and function, and their silencing alters centrosome and spindle pole numbers, frequently producing multipolar spindles and defects in chromosome segregation and nuclear morphology.^15^ To further validate the functional relevance of the identified *VSP4A* mutations, we analyzed centrosome and mitotic spindle organization in synchronized proband-derived fibroblasts carrying the heterozygous p.Arg284Trp or p.Arg284Gly substitutions. As expected, in interphase, control cells typically had two discernible centrosomes (Figure 6A). Similarly, during mitosis, these cells formed normal bipolar spindles with two centrosomes. In metaphase, canonical mitotic spindles with properly aligned chromosomes were observed in the vast majority of cases (Figure 6B). In contrast, proband fibroblasts showed an anomalous centrosome number and morphology in interphase (Figure 6A); similarly, multipolar spindles were observed during mitosis, resulting in a high frequency of aberrant chromosome alignment during metaphase (Figure 6B). Aberrant chromosome segregation was documented by the presence of both lagging and bridging chromosomes during anaphase and telophase (Figure 6B) and was associated with polyploidy and production of micronuclei, i.e., encapsulated lagging chromosomes or damaged chromosome fragments not incorporated in the main nucleus after anaphase (Figure 7A). Consistent with these findings, a high proportion of proband fibroblasts were observed at G2/M, as measured by BrdU incorporation flow cytometry analysis (Figure 7B), which possibly results from altered G2/M and abscission checkpoint activation and/or a faulty progression toward cell division.^48^, ^49^, ^50^

### Proband Fibroblasts Have Abnormal Nuclear Envelope Morphology and Increased DNA Damage

**Figure 8:**
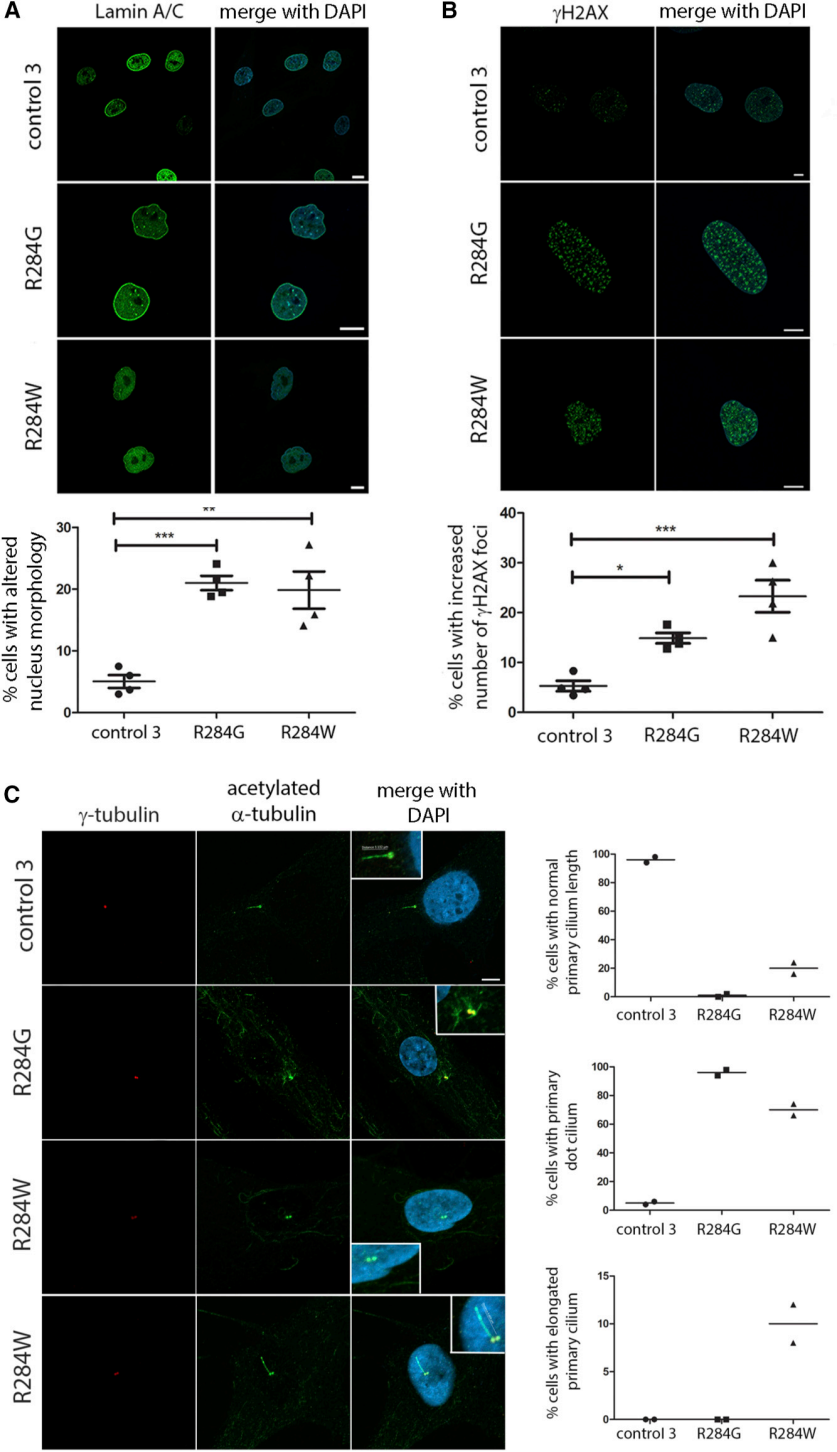
Proband Fibroblasts Have Abnormal Nuclear Envelope Morphology, Increased DNA Damage, and Abnormal Primary Cilium Morphology (A) An increased number of aberrantly shaped nuclei in fibroblasts carrying the *VPS4A* mutations was observed versus control cells. Staining was performed using anti-lamin A/C and DAPI. Scale bar is 10 μm. (B) Representative images showing an increase number of γ-H2AX foci in probands’ fibroblasts carrying compared to control cells. The staining was performed using γ-H2AX antibody and DAPI. Scale bar is 2.5 μm. In all experiments, mean ± SEM of 4 separate counts (≥40 cells/line each) for a total of 200 cells/line scored. p values were calculated by one-way ANOVA with Tukey’s correction for multiple testing. (C) Confocal images showing altered primary cilium morphology in proband fibroblasts compared to control cells. Cells heterozygous for the p.Arg284Gly amino acid change show absent cilia with only a visible basal body (dot cilium, zoomed image), whereas cells with the p.Arg284Trp substitution show either a dot cilium or occasionally an elongated or normal cilium (zoomed image). Primary cilia are labeled with acetylated α-tubulin, basal bodies and nuclei are labeled with γ-tubulin and DAPI, respectively. Scale bar is 5 μm. 100 cells were analyzed for each line over two independent experiments, bars represent the mean.

During anaphase, when chromosome separation has been achieved, the nuclear envelope is reassembled around the forming nuclei, to coordinate proper segregation of the nuclear content in daughter cells and assure the structural integrity and functionality of the nuclear compartment. The ESCRT-III complex and VPS4A and B contribute to nuclear envelope sealing and spindle disassembly at the nuclear envelope-microtubule intersection sites during mitotic exit,^51^^,^^52^ and defective ESCRT function causes abnormal nuclear membrane sealing and altered morphology.^53^^,^^54^ Moreover, the ESCRT-III complex contributes to repair of nuclear envelope ruptures during interphase, and expression of a dominant-negative VPS4A protein delayed repair.^55^^,^^56^ Based on these considerations, we explored possible changes in nuclear morphology and architecture in proband fibroblasts expressing the VPS4A-p.Arg284Trp or -p.Arg284Gly mutants. Immunofluorescence microscopy with the nuclear envelope marker lamin A/C labeling demonstrated a significant increase in the proportion of proband-derived cells with irregular nuclear morphology compared to control cells (Figure 8A). It has been established that nuclear deformation may result in a breaking of the nuclear envelope, which in turn exposes chromosomal DNA to the cytoplasmic environment, thus promoting DNA damage. Consistent with this, immunofluorescence microscopic identification of γH2AX foci, a marker of damaged DNA, showed an increased number of positive cells and foci per cell among those carrying the VSP4A mutants compared to control cells (Figure 8B), indicating increased spontaneous DNA damage. Thus nuclear envelope morphology and integrity are altered in fibroblasts from individuals with heterozygous VPS4A mutations.

### Defective VPS4A Function Affects Primary Cilium Morphology

Vesicular trafficking plays an essential role in cilium biogenesis and function. Of note, VPS4 has been identified as a dynamic component of the ciliary transition zone, a region in which the mother centriole, tethering to plasma membrane by the transition fibers, becomes the basal body for primary cilium formation. Defective VPS4A ATP hydrolysis causes a block of ciliogenesis after formation of the ciliary vesicle and this function appears to be ESCRT-III independent.^16^ We therefore analyzed primary cilium biogenesis and morphology in fibroblasts from affected subjects. Assessment of cilium structure in starved fibroblasts revealed the presence of aberrant primary cilium formation in both fibroblast lines with mutated *VPS4A* alleles. Specifically, normal cilia were absent in fibroblasts expressing the VPS4A-p.Arg284Gly mutant, which instead showed a visible basal body (dot cilium); similarly, a dot cilium was documented in most fibroblasts heterozygous for the p.Arg284Trp substitution, although a small number of elongated cilia were also observed in cells from this proband (Figure 8C). Thus, defective VPS4A has pleiotropic consequences on diverse cellular processes, including perturbation of a variety of centrosome-dependent structures.

## Discussion

The ESCRT-III complex and VPS4 together form a multifunctional membrane modeling machinery. Against the background of the multiplicity of functions ascribed to ESCRT-III, it is perhaps surprising that only three complex members, CHMP1A, CHMP2B, and CHMP4B, have been implicated in Mendelian genetic disease thus far.^17^, ^18^, ^19^ This may be explained by functional redundancy between ESCRT-III complex components, or because loss of components might lead to an embryonic lethal phenotype, as has been observed in some, but not all, mouse models lacking specific CHMP proteins.^57^^,^^58^ However, we now report a distinct syndromic neurodevelopmental disorder caused by dominantly acting amino acid substitutions in VPS4A, a key enzyme that regulates ESCRT-III function. We propose the acronym CIMDAG (cerebellar hypoplasia and cataracts, intellectual disability, congenital microcephaly, dystonia and dyserythropoeitic anemia, growth retardation) to highlight the main clinical features of this syndrome, which may also include other structural brain abnormalities, retinal dystrophy, hepatosplenomegaly, and sensorineural deafness. The hematological features of this condition are thoroughly characterized in the accompanying paper.^59^

The pleotropic clinical effects observed in CIMDAG likely reflect the multitude of cellular functions in which ESCRT-III and VPS4A participate, including those demonstrated to be affected by our studies. In the future it will be important to unravel which ESCRT-III and VPS4A functions underlie the pathology of the different clinical phenotypes we observed. The centrosomal and mitotic defects we observed are strong candidates to underlie microcephaly and other growth impairments in CIMDAG. These processes play a crucial role during brain development, including in neurogenesis, neuronal migration, and polarity, and defects in them commonly underlie neurodevelopmental diseases.^60^ Defects in mitosis have also been linked to dyserythropoietic anemia and are a feature of the anemia seen in CIMDAG.^59^^,^^61^ In contrast, congenital cataract is a recognized feature of lysosomal storage diseases and so it may be related to the endolysosomal dysfunction that we observed, which may also be relevant for the health of mature post-mitotic neurons, as it has been implicated in several forms of neurodegeneration.^62^ Finally, it is striking that many of the clinical features found in our probands (including cerebellar hypoplasia, retinal dystrophy, Leber amaurosis, DD, ID, cataract, sensorineural deafness, and hypogonadism) occur in ciliopathies, and our data support the proposed role of VPS4A in controlling primary cilium morphogenesis.^16^^,^^63^ Thus it is possible that abnormal primary cilium function contributes to the CIMDAG phenotype.

Multiple heterozygous *VPS4A* loss-of-function mutations are present in general population databases, indicating that a haploinsufficiency mechanism is unlikely to cause the type of severe early childhood condition that we describe.^64^ In contrast, our data and published evidence point to the p.Glu206Lys, p.Arg284Trp, and p.Arg284Gly mutants having a dominant-negative effect. Overexpression of each of these mutants in immortalized cells caused development of the highly characteristic enlarged endosomal structures that are induced by expression of known dominant-negative forms of VPS4A. Similar, although less marked, enlarged endosomal phenotypes were also observed in proband cells. As VPS4A protein expression was not altered in proband cells, assuming equal expression of wild-type and mutant VPS4A we expect that a large majority of VPS4A hexamers will have impaired function as they will contain at least one mutant subunit. This may explain the similar endosomal phenotypes we observed in proband fibroblasts and iPSC-derived neurons lacking VPS4A.

The archetypal function for VPS4 proteins is in endosomal sorting and our observations elucidate details of the physiological role of VPS4A in this process. Overexpression of dominant-negative VPS4A in cultured cells affects ILV formation, causes trapping of ESCRT proteins on the endosomal membrane, and inhibits EGFR degradation.^37^^,^^38^ However, a surprising observation in our study was that proband fibroblasts expressing dominant-negative *VPS4A* mutations at heterozygous physiological levels showed no obvious effect on ILV formation or EGFR degradation. Consistent with this, we did not observe accumulation of the “core” ESCRT-III complex member CHMP2B on the endosomal membrane, suggesting that functional VPS4A is not required for removal of the core ESCRT-III complex from the endosomal membrane. This may be because of redundancy between VPS4A and VPS4B—previous studies have shown that double knock-down of these proteins is required for formation of a “VPS4-dominant-negative”-type endosomal compartment. An ESCRT-independent ILV formation pathway has been described, which could also provide an explanation for retained ILV formation.^65^^,^^66^ In contrast, the endosomal localization of IST1 was increased in both proband fibroblasts and iPSC-derived neurons lacking VPS4A, so it appears that VPS4A is absolutely required for recycling of this protein, the best-characterized function of which is in promoting endosomal tubule fission.^5^^,^^6^ Further studies will be required to elucidate the functional consequences of this accumulation on endosomal tubule fission dynamics and endosomal receptor traffic, and its relationship to pathogenesis in our probands, but we speculate that the enhanced EGFR degradation we observed in patient fibroblasts may be explained by defective tubular sorting of this receptor away from endosomal degradation.

In summary, we have identified *de novo* missense mutations affecting the key ESCRT-regulation enzyme VPS4A in probands with a distinct multisystem neurodevelopmental condition. Study of the functional effects of the mutations demonstrated that they act by a dominant-negative mechanism to cause effects on multiple ESCRT-dependent cellular pathways and indicate an absolute requirement for proper VPS4A function in neurodevelopment and other physiological developmental processes in humans.

## Data and Code Availability

The pathogenic variants identified in this work have been submitted to ClinVar (submission ID: SUB7923752). WES and WGS datasets have not been deposited in a public repository due to privacy and ethical restrictions but are available from the corresponding authors on request. The 100,000 Genomes Project data used in this work are available from the Genomics England Research Environment, with restrictions to ensure the confidentiality and appropriate use of the data.

## Consortia

Members of the Genomics England Research Consortium are as follows: J.C. Ambrose, P. Arumugam, E.L. Baple, M. Bleda, F. Boardman-Pretty, J.M. Boissiere, C.R. Boustred, H. Brittain, M.J. Caulfield, G.C. Chan, C.E.H. Craig, L.C. Daugherty, A. de Burca, A. Devereau, G. Elgar, R.E. Foulger, T. Fowler, P. Furio-Tari, J.M. Hackett, D. Halai, A. Hamblin, S. Henderson, J.E. Holman, T.J.P. Hubbard, K. Ibanez, R. Jackson, L.J. Jones, D. Kasperaviciute, M. Kayikci, L. Lahnstein, K. Lawson, S.E.A. Leigh, I.U.S. Leong, F.J. Lopez, F. Maleady-Crowe, J. Mason, E.M. McDonagh, L. Moutsianas, M. Mueller, N. Murugaesu, A.C. Need, C.A. Odhams, C. Patch, D. Perez-Gil, D. Polychronopoulos, J. Pullinger, T. Rahim, A. Rendon, P. Riesgo-Ferreiro, T. Rogers, M. Ryten, K. Savage, K. Sawant, R.H. Scott, A. Siddiq, A. Sieghart, D. Smedley, K.R. Smith, A. Sosinsky, W. Spooner, H.E. Stevens, A. Stuckey, R. Sultana, E.R.A. Thomas, S.R. Thompson, C. Tregidgo, A. Tucci, E. Walsh, S.A. Watters, M.J. Welland, E. Williams, K. Witkowska, S.M. Wood, and M. Zarowiecki.

## Declaration of Interests

The authors declare no competing interests.

## Web Resources

Addgene, http://www.addgene.org/
ClinVar, https://www.ncbi.nlm.nih.gov/clinvar/
dbNSFP, https://sites.google.com/site/jpopgen/dbNSFP
ExAC database, http://exac.broadinstitute.org/
gnomAD, https://gnomad.broadinstitute.org/
NHLBI Trans-Omics for Precision Medicine (TOPMed), https://www.nhlbiwgs.org/
Online Mendelian Inheritance in Man (OMIM): http://www.omim.org
Pfam, https://pfam.xfam.org/
Protein Data Bank (PDB), https://www.rcsb.org/
UniProtKB, https://www.uniprot.org/

## Supplemental Information

Document S1. Supplemental Note, Supplemental Material and Methods, Consortium Membership List, Supplemental Figures S1–S8, and Table S1

Document S2. Article plus Supplemental Information
