# Supplementary material for: De Novo VPS4A Mutations Cause Multisystem Disease with Abnormal Neurodevelopment
Source: Am J Hum Genet. 2020 Nov 12;107(6):1129–48. doi: 10.1016/j.ajhg.2020.10.012 (PMC7820634; doi:10.1016/j.ajhg.2020.10.012)
Supplement: Document S1. Supplemental Note, Supplemental Material and Methods, Consortium Membership List, Supplemental Figures S1–S8, and Table S1 [file mmc1.pdf]

The American Journal of Human Genetics, Volume *107*

Supplemental Data

De Novo VPS4A Mutations Cause Multisystem Disease with Abnormal Neurodevelopment

Catherine Rodger, Elisabetta Flex, Rachel J. Allison, Alba Sanchis-Juan, Marcia A. Hasenahuer, Serena Cecchetti, Courtney E. French, James R. Edgar, Giovanna Carpentieri, Andrea Ciolfi, Francesca Pantaleoni, Alessandro Bruselles, Genomics England Research Consortium, Roberta Onesimo, Giuseppe Zampino, Francesca Marcon, Ester Siniscalchi, Melissa Lees, Deepa Krishnakumar, Emma McCann, Dragana Yosifova, Joanna Jarvis, Michael C. Kruer, Warren Marks, Jonathan Campbell, Louise E. Allen, Stefano Gustincich, F. Lucy Raymond, Marco Tartaglia, and Evan Reid

Supplemental Note: Case Reports for representative probands.

Proband 1 (c.850A>T, p.Arg284Trp).

Proband 1 is the 1st child of healthy unrelated parents. Her father was 35 years old and mother 33 years old at the time of her birth. Routine antenatal testing showed a low PAPP-A; a Harmony trisomy screening test was normal. No abnormalities were noted on antenatal ultrasound scan, but in view of the low PAPP-A, serial ultrasound screening was undertaken; the appearances were normal until around the 36 weeks scan, when growth was noted to be small. A semi-urgent caesarean section was undertaken in view of the growth and breech presentation; she was born with birthweight of 2310g (Z -2.8) with a head circumference of 31.4cm (Z -2.6) at 37 weeks.

She cried at birth, and did not need any active resuscitation. She would not breast-feed but sucked reasonably well by bottle with expressed breast milk. She failed her newborn hearing test twice, but further testing subsequently confirmed normal hearing.

Parents were concerned about her visual behaviour and that she was not fixing or following at 8 weeks of age. Ophthalmological examination showed significant central and lamellar lens opacities but no microphthalmia or anterior segment abnormalities. Her father at that time was found to have very mild symptomatic bilateral sutural lens opacities. Following cataract removal, fundal examination on the proband showed an abnormal retinal appearance with abnormal atrophic changes at the macula in keeping with a retinal dystrophy, which was confirmed with very poor ERG and VEP amplitudes.

She developed mild symptoms of cow's milk protein intolerance and gastro-oesophageal reflux, which was treated with ranitidine and omeprazole, and started on Nutramigen. Feeding has continued to be a problem; at the age of 2 years she takes no solids orally, and milk is mild given via NGT. She has persistent vomiting and poor weight gain.

Developmental progress has been slow. She started smiling from around 6 months of age, around which time head control was improving; she was beginning to move her head to sounds at 7 months of age. At the age of 2 years, she is able to roll from back to front, and pivot on her back using her legs, but is not able to support her head on her hands when prone. She is not sitting independently. She laughs responsively and is able to attract

parents' attention by laughing. She recognises her parents' voices, and will respond to familiar stories and songs. She has no words, but has some vocalisations.

Sleep pattern is poor. She developed obstructive sleep apnoea, and underwent a tonsillectomy. She takes melatonin and tends to fall asleep easily, but will often wake after 30-60 minutes and then repeatedly throughout the night, sometimes staying awake for several hours.

Examination showed central hypotonia, with hands held in a clenched position. She tends to hold her mouth open with a protruding tongue. Her movements are dyskinetic. She has small hands and feet.

Array CGH, neurometabolic investigations and a TORCH screen were normal. An echocardiogram was reported as normal. NGS of a panel of genes associated with cataracts and retinal dystrophy did not identify any pathogenic changes. She has had an anaemia with haemoglobin of 88 g/l in infancy, then later 83 g/l, but possibly dietary owing to her intake. The blood film stated that the red cells showed anisocytosis.

MRI: Sulci are mildly prominent which in conjunction with microcephaly suggest cerebral volume loss. The corpus callosum is present; it is thin suggesting white matter volume loss. Sylvian fissures are widened anteriorly. Cortical gyration is likely to be within normal limits. Cerebellar vermis and hemispheres are hypoplastic and there is a small posterior fossa. Brain stem is within normal limits.

ERG showed no consistent retinal responses above the level of noise to a range of stimulus intensities.

VEPs showed no consistent pattern reversal cortical responses evident above the level of noise confirming a very degraded pattern.

<u>Proband 2 (c.850A>G, p.Arg284Gly).</u>

An 8-month-old male presented with a phenotype characterized by microcephaly (-5 SD), congenital cataract, large ears, long palpebral fissures, strabismus, retrognathia with wide mandibular angle, single transverse palmar crease, bilateral clubfoot, hypertonia, global developmental delay. The pregnancy was uncomplicated. He was born to partners who were cousins, at 39 weeks of gestation, by elective secondary caesarean section. Auxologic

parameters were normal, Apgar score was 3 at 1 minute and 9 at 5 minutes after birth, orotracheal intubation was not performed.

Four days after birth he developed severe haemolytic anaemia requiring transfusion. Haemolytic episodes recurred leading to liver and spleen enlargement, common causes of anaemia and haemolisis were ruled out. Afterwards the proband's medical history was significant for severe oromotor disability and failure to thrive with weight and length < -5 SD, parents declined artificial nutrition. He presented with hypovision and moderate bilateral sensorineural deafness.

At 18 months psychomotor development delay was confirmed, cerebral MRI revealed corpus callosum hypoplasia and severe cerebellar hypoplasia. Electroencephalography detected abnormal brain electrical activity; the proband didn't present with clinical seizures. The neurological pattern got worse and he developed severe intellectual disability and total dependence from caregiver.

At the last clinical evaluation, 3 months before death, he presented with severe malnutrition, microcephaly, large ears, long palpebral fissures, strabismus, long oval face, long philtrum, gingival hypertrophy, exposed upper incisors, retrognathia with wide mandibular angle, single transverse palmar crease, flexion deformity of wrist, elbow, knee with popliteal pterigium, bilateral clubfoot, scoliosis of the spine and hypogenitalism. He also showed yellow sclera, suggestive of liver involvement. He died at the age of 29 years from respiratory failure secondary to pneumonia.

Proband 3 (c.850A>T, p.Arg284Trp).

The proband was the second child to unrelated Caucasian parents. He was born at term following an uncomplicated pregnancy and discharged home at 6 hours of age. He was readmitted within 24 hours with vomiting and drowsiness. He was investigated for sepsis and treated with antibiotics. Microcephaly and hypotonia were noted on that admission. He continued to vomit and had failure to thrive. Feeding became a significant issue and never was successfully established – he had a PEG inserted but vomiting remained problematic. He subsequently had a fundoplication and a jejunal tube placed – this helped his symptoms but he continued to retch. He made very little in the way of developmental progress – he could hold his head in the midline and responded to his parents voice; he never fixed and

followed. From the beginning, he had episodes of arching and irritability – initially thought to be due to significant gastro-oesophageal reflux. With time, these evolved into dystonic and dyskinetic movements. As his condition progressed, he was diagnosed with 'salt and pepper' retinitis, cataract and liver dysfunction. He had progressively enlarging liver and mild conjugated hyperbilirubinaemia and abnormal liver function tests. He did not develop liver failure. Prior to his death he had short episodes of gazing in one direction associated with limb movements but was never formally diagnosed with seizures. He died at 26 months of age.

He was extensively investigated from a neurometabolic point of view and his positive findings included

1. MRI - bilateral frontal polymicrogyria and Pontocerebellar hypoplasia
2. CK – raised on 2 occasions to 1500, normal on other occasions
3. Muscle Biopsy – said to show features of myopathy
4. Liver Biopsy – microvesicular steatosis and haemosiderosis
5. Respiratory chain analysis – normal
6. Microarray 17p11.2 del - VUS paternally inherited
7. Cardiff Cortical malformation gene panel– no significant findings

Additional findings at Post mortem included

1. Skeletal survey showed 'hair on end' appearance to outer table of skull
2. Moderate abdominal ascites
3. Malrotation and volvulus - Appendix, caecum and proximal ascending colon in left upper quadrant

The clinical impression was always of a rare neuro metabolic disorder.

Author List for Genomics England Research Consortium

Ambrose J. C. [1], Arumugam P.[1], Baple E. L. [1], Bleda M. [1], Boardman-Pretty F. [1,2], Boissiere J. M. [1], Boustred C. R. [1], Brittain H.[1], Caulfield M. J.[1,2], Chan G. C. [1], Craig C. E. H. [1], Daugherty L. C. [1], de Burca A. [1], Devereau, A. [1], Elgar G. [1,2], Foulger R. E. [1], Fowler T. [1], Furió-Tarí P. [1], Hackett J. M. [1], Halai D. [1], Hamblin A.[1], Henderson S.[1,2], Holman J. E. [1], Hubbard T. J. P. [1], Ibáñez K.[1,2], Jackson R. [1], Jones L. J. [1,2], Kasperaviciute D. [1,2], Kayikci M. [1], Lahnstein L. [1], Lawson K. [1], Leigh S. E. A. [1], Leong I. U. S. [1], Lopez F. J.[1], Maleady-Crowe F. [1], Mason J. [1], McDonagh E. M. [1,2], Moutsianas L. [1,2], Mueller M. [1,2], Murugaesu N. [1], Need A. C. [1,2], Odhams C. A. [1], Patch C. [1,2], Perez-Gil D. [1], Polychronopoulos D. [1], Pullinger J. [1], Rahim T. [1], Rendon A. [1], Riesgo-Ferreiro P.[1], Rogers T. [1], Ryten M. [1], Savage K. [1], Sawant K. [1], Scott R. H. [1], Siddiq A. [1], Sieghart A. [1], Smedley D. [1,2], Smith K. R. [1,2], Sosinsky A. [1,2], Spooner W. [1], Stevens H. E. [1], Stuckey A. [1], Sultana R. [1], Thomas E. R. A. [1,2], Thompson S. R. [1], Tregidgo C. [1], Tucci A. [1,2], Walsh E. [1], Watters, S. A. [1], Welland M. J. [1], Williams E. [1], Witkowska K. [1,2], Wood S. M. [1,2], Zarowiecki M.[1]

1. Genomics England, London, UK
2. William Harvey Research Institute, Queen Mary University of London, London, EC1M 6BQ, UK.

Supplemental Methods

EGFR degradation assay

EGFR degradation assays were performed as described previously.[32] Briefly, proband fibroblasts were serum starved overnight before the addition of 100 ng/mL EGF (Calbiochem) in the presence of 10 µg/mL cyclohexamide (Sigma-Aldrich). Cells were lysed at 0, 30, 90 and 180 minute time-points, then analysed by western blotting.

Electron Microscopy

Fibroblasts were seeded to Thermanox (Thermo Fisher Scientific) plastic coverslips and fixed with 2% PFA, 2.5% glutaraldehyde, and 0.1 M cacodylate buffer (pH 7.2). Cells were post-fixed with 1% osmium tetroxide:1.5% potassium ferricyanide before being incubated with 1% tannic acid to enhance contrast. Cells were dehydrated using increasing percentages of ethanol before being embedded onto EPON stubs or beam capsules. Resin was cured overnight at 65°C, and coverslips were removed using a heat-block. Ultrathin (50- to 70-nm) conventional sections were cut using a diamond knife mounted to a Reichart ultracut S ultramicrotome. Sections were collected onto copper grids stained using lead citrate. Sections were viewed on a FEI Tecnai transmission electron microscope at a working voltage of 80 kV.

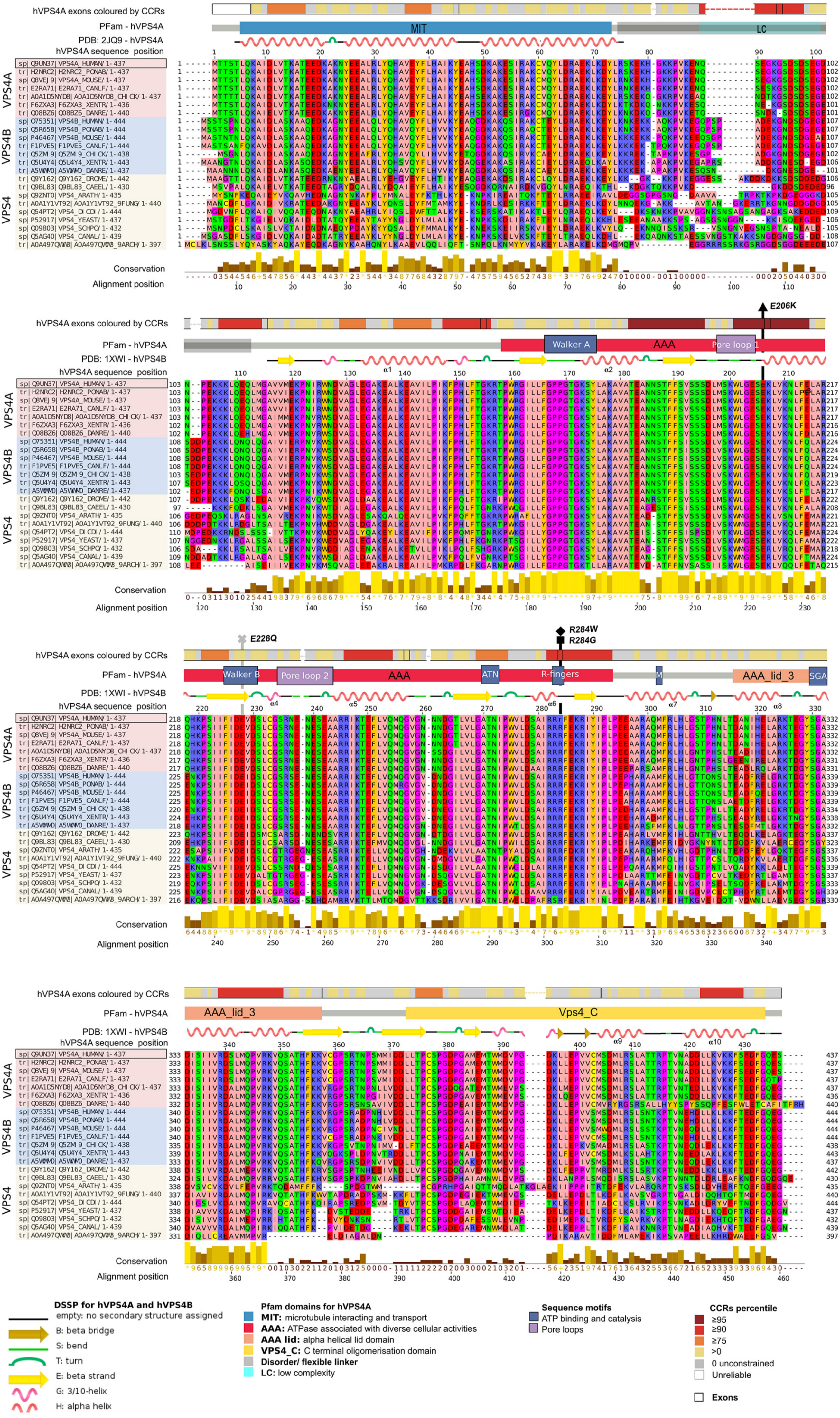
hVPS4A exons coloured by CCRs
PFam - hVPS4A
PDB: 2JQ9 - hVPS4A
hVPS4A sequence position
PDB: 1XWI - hVPS4B
MIT
LC
Walker A
AAA
Pore loop 1
E206K
E228Q
R284W
R284G
Walker B
Pore loop 2
ATN
R-fingers
AAA_lid_3
SGA
Vps4_C
VPS4A
VPS4B
VPS4
Conservation
Alignment position
DSSP for hVPS4A and hVPS4B
empty: no secondary structure assigned
B: beta bridge
S: bend
T: turn
E: beta strand
G: 3/10-helix
H: alpha helix
Pfam domains for hVPS4A
MIT: microtubule interacting and transport
AAA: ATPase associated with diverse cellular activities
AAA lid: alpha helical lid domain
VPS4_C: C terminal oligomerisation domain
Disorder/ flexible linker
LC: low complexity
Sequence motifs
ATP binding and catalysis
Pore loops
CCRs percentile
≥95
≥90
≥75
>0
0 unconstrained
Unreliable
Exons

Figure S1

Figure S1. Multiple sequence alignment of VPS4 proteins from representative species. The novel *de novo* missense variants in VPS4A are represented in the corresponding columns of the alignment, using the same symbols as in Figure 1. The top track depicts CCRs percentiles for human VPS4A (hVPS4A). The protein domains depicted in the second track are based on hVPS4A (UniProtKB entry Q9UN37). The third track shows secondary structural elements based on PDB structures for different regions of hVPS4A or human VPS4B (hVPS4B). In the multiple sequence alignment residues are coloured according to their physicochemical properties (Jalview color scheme). PONAB= orangutan; MOUSE= mouse; CANLF= dog; CHICK= chicken; XENTR= frog; DANRE= zebrafish; DROME= drosophila; CAEEL= worm; ARATH= arabidopsis; 9FUNG= fungus; DICDI= slime mold; SCHPO= yeast; CANAL= Candida albicans; 9ARCH= heimdallarchaeota. The bottom track shows amino acid conservation.

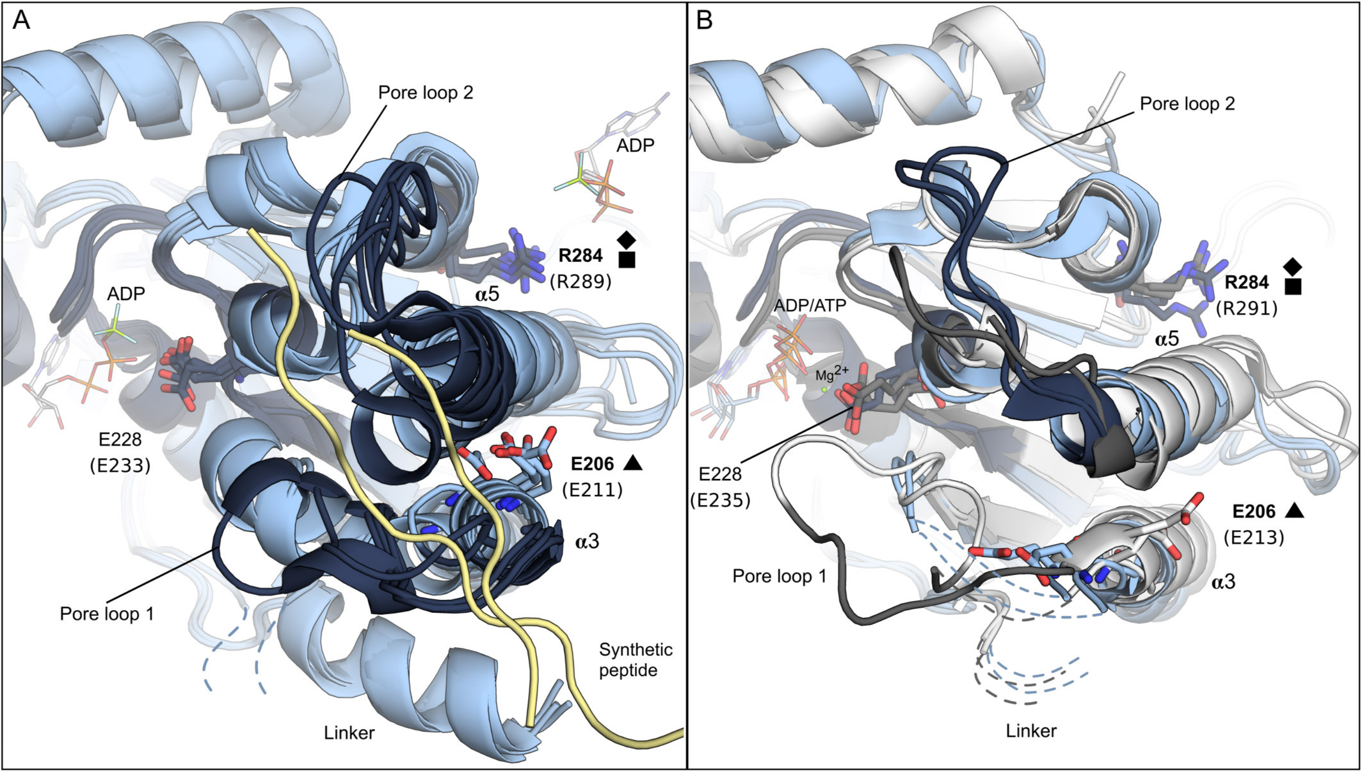
A
Pore loop 2
ADP
R284
(R289)
α5
ADP
E228
(E233)
E206
(E211)
α3
Pore loop 1
Synthetic
peptide
Linker
B
Pore loop 2
R284
(R291)
α5
ADP/ATP
Mg2+
E228
(E235)
E206
(E213)
α3
Pore loop 1
Linker

Figure S2

Figure S2. Protein structural superposition of the ATPase domain of A) the 6 chains of the VPS4 hexamer from yeast (PDB ID: 6OO2) and B) the monomeric VPS4B from human (PDB ID: 1XWI) and mouse (PDB ID: 2ZAM, 2ZAO, 2ZAN). In (**B**), chains in white correspond to apo structures and chains in blue have ADP or ATP bound. The superposition of all chains was done using the human structure as reference. In yellow is the synthetic peptide that shows how the ESCRT-III proteins would translocate through the pore. Residues with missing coordinates are represented with dashed lines. The flexible linker connector is missing in human and mouse structures (**B**) but can be partially observed as α-helix in four chains of yeast VPS4 structure (**A**).

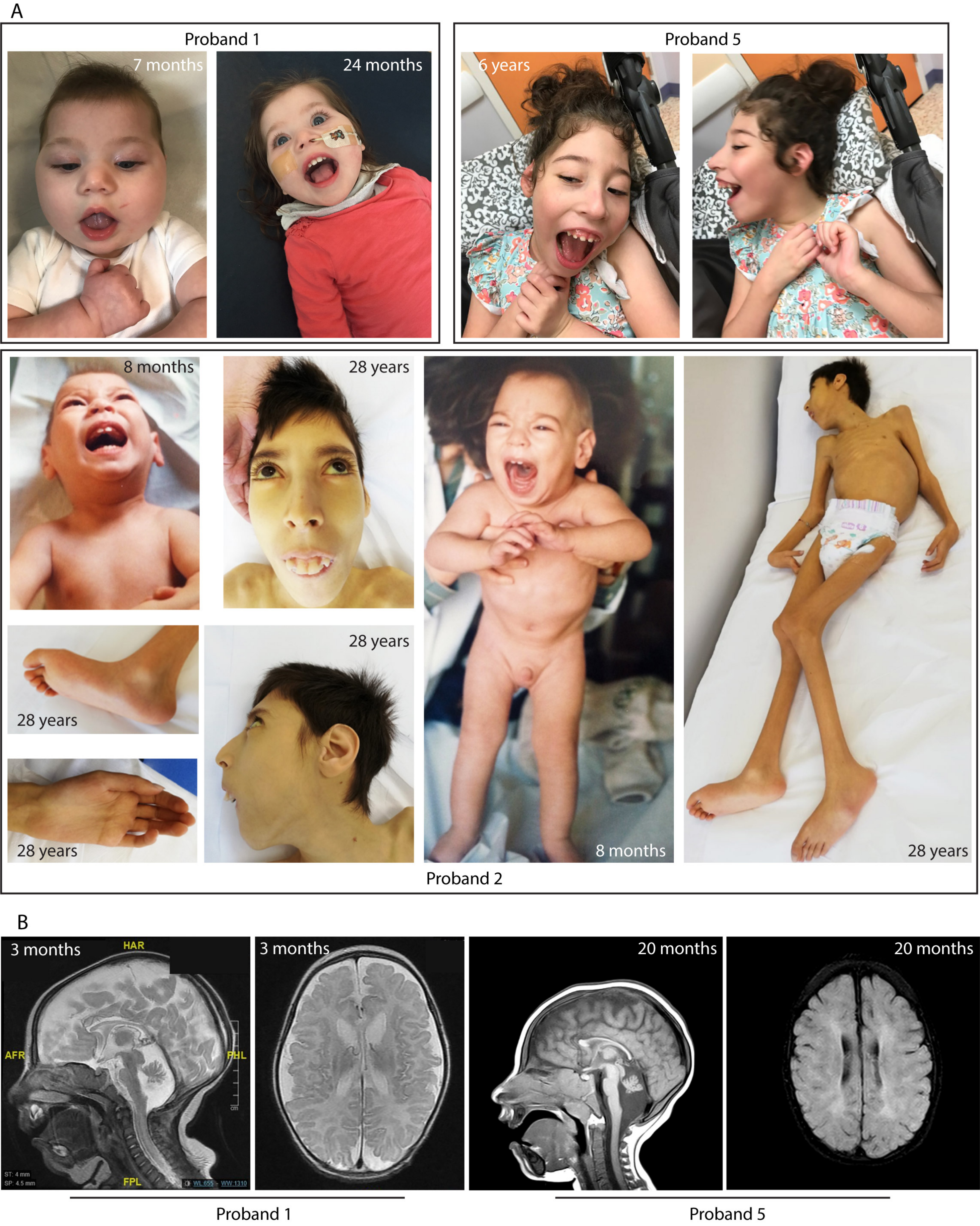
A
Proband 1
7 months
24 months
Proband 5
6 years
8 months
28 years
28 years
28 years
28 years
8 months
28 years
Proband 2
B
3 months
HAR
AFR
PHL
FPL
3 months
20 months
20 months
Proband 1
Proband 5

Figure S3

Figure S3. Additional clinical images of probands. A) Photographs showing probands 1, 2 and 5. Note common features of microcephaly, long palperal fissures, strabismus, long relatively smooth philtrum and broad and arched eyebrows. In the older probands, large ears, long oval face, gingival hypertrophy, exposed upper incisors and retrognathia with wide mandibular angle are common features. Proband 2 also had jaundice, a single transverse palmar crease, flexion of wrist, elbow, knee with popliteal pterigium, bilateral clubfoot and scoliosis. **B**) MRI images of probands 1 and 5. Proband 1: Sagittal and axial T2 MRI images, Proband 5, left is sagittal T1 MRI image and right is T2 flair axial image. Abnormal features in both include thinning of the corpus callosum, atrophy or under-development of the cerebellum, pontine hypoplasia and white matter volume loss. In proband 1 there is extensive dysgyria and in proband 5 there is periventricular gliosis and scalloping of the lateral ventricles.

A

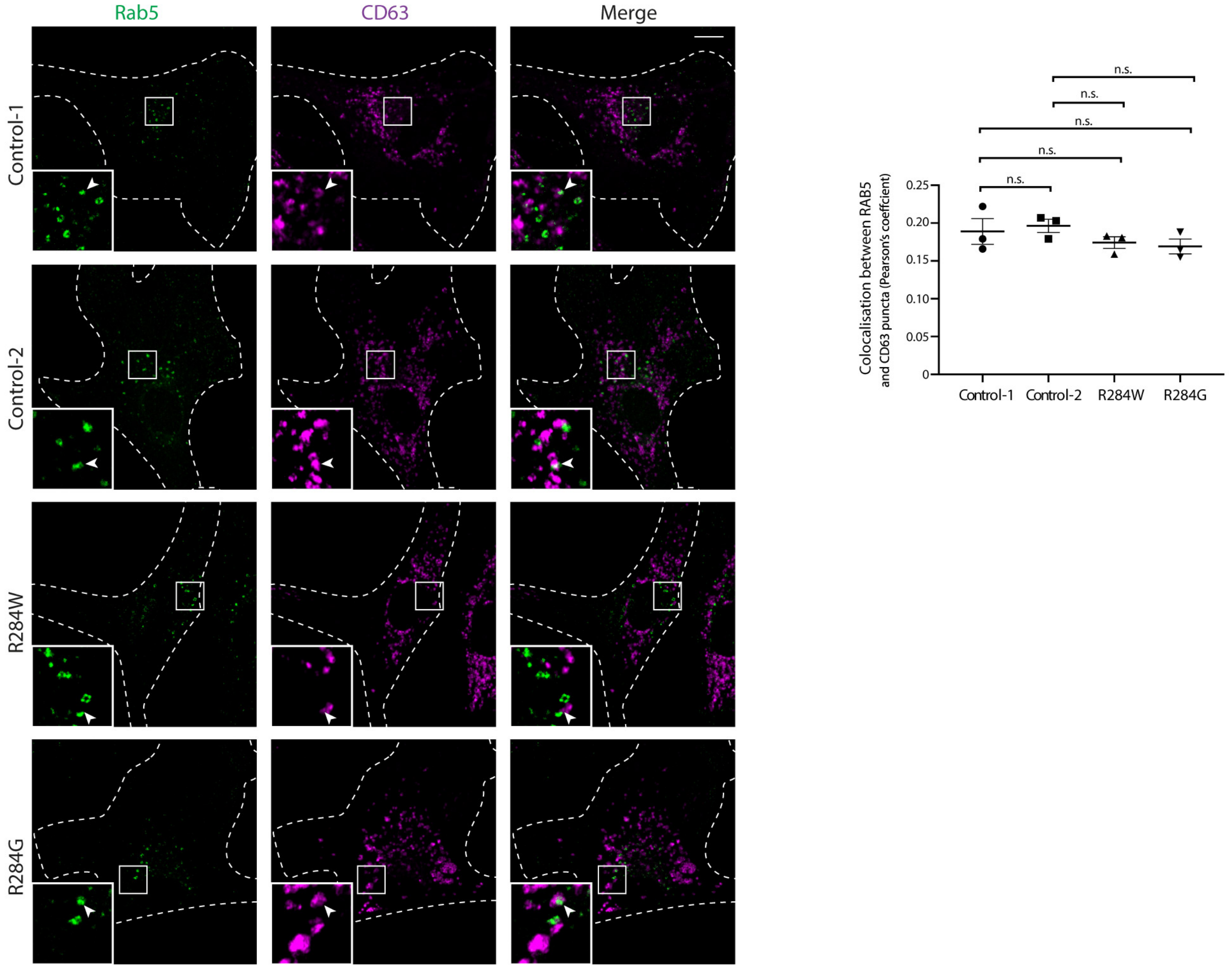

B

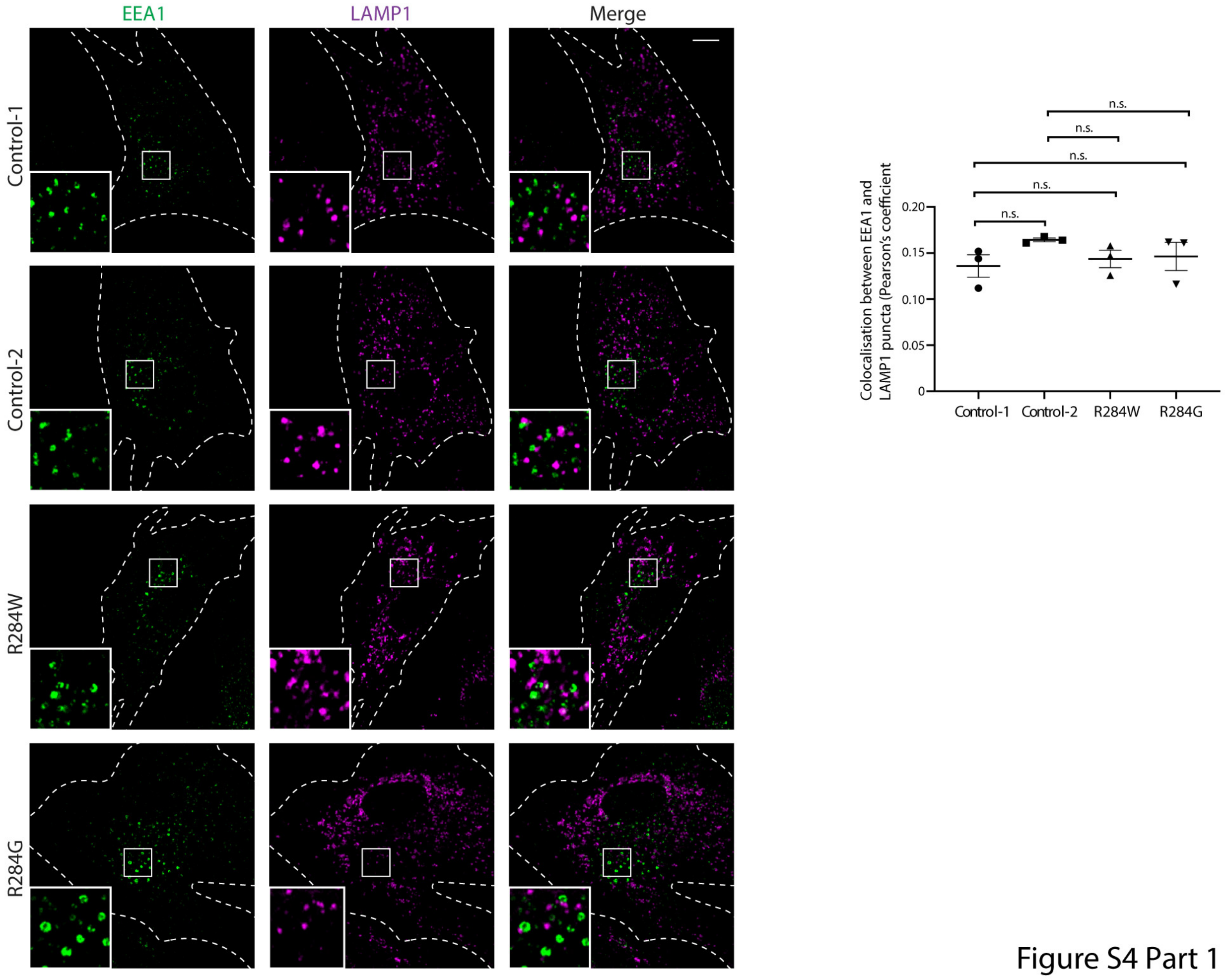

Figure S4 Part 1

C

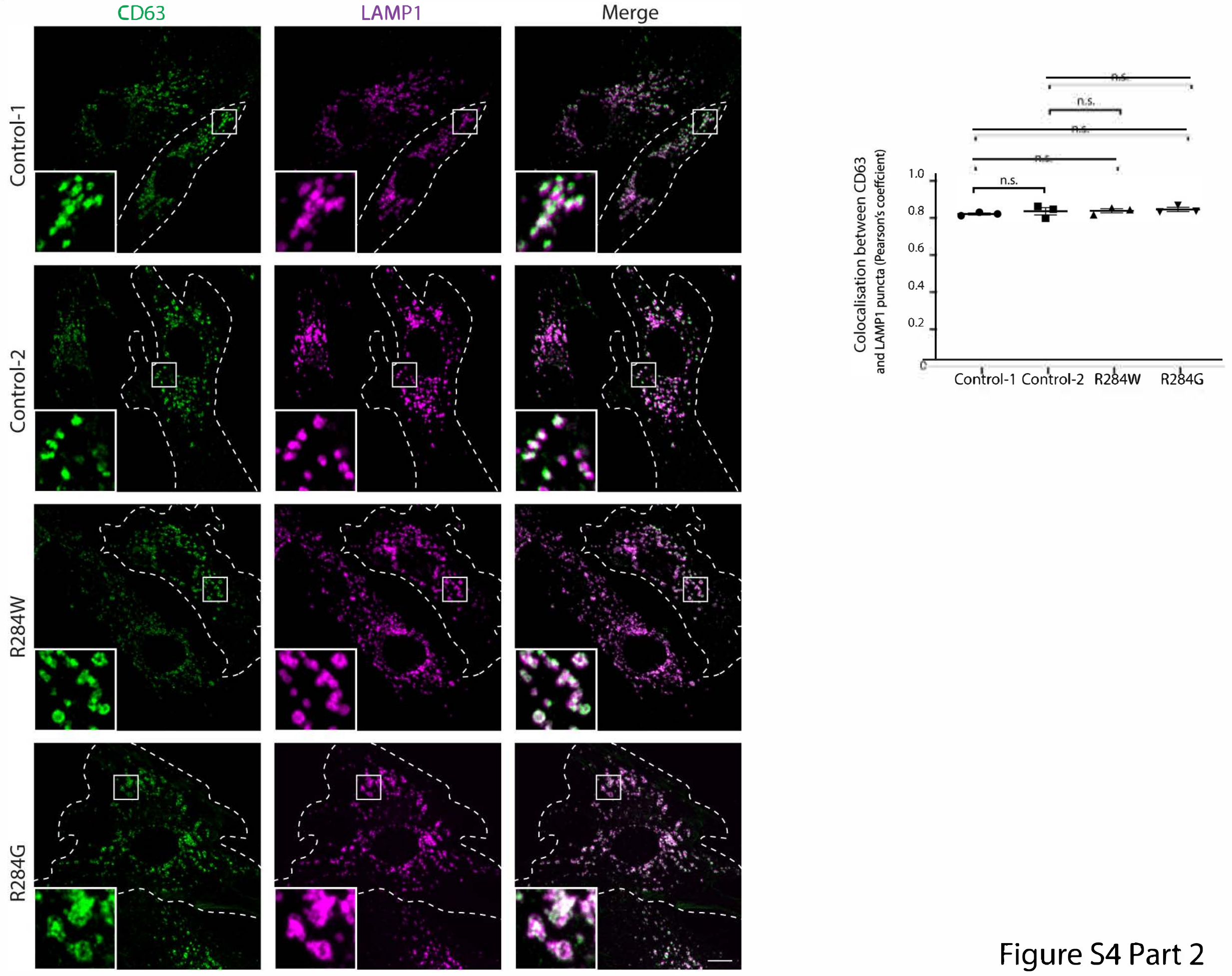

Figure S4 Part 2

Figure S4. VPS4A proband fibroblasts show no endosomal compartment content mixing. Cultured fibroblasts from control subjects and the probands indicated were fixed, labelled with the early endosomal marker RAB5 and CD63 (**A**), EEA1 and LAMP1 (**B**) and CD63 and LAMP1 (**C**), then visualised by confocal immunofluorescence microscopy. Colocalisation was measured using Pearson's co-efficient (in 20 cells in each repeat), and the result of n=3 biological repeats for each set of markers is quantified in the corresponding charts. Bars indicate mean ± S.E.M., p-values were calculated by one-way ANOVA with Tukey's post-hoc test for repeated measures. Micrograph scale bars = 10 μm.

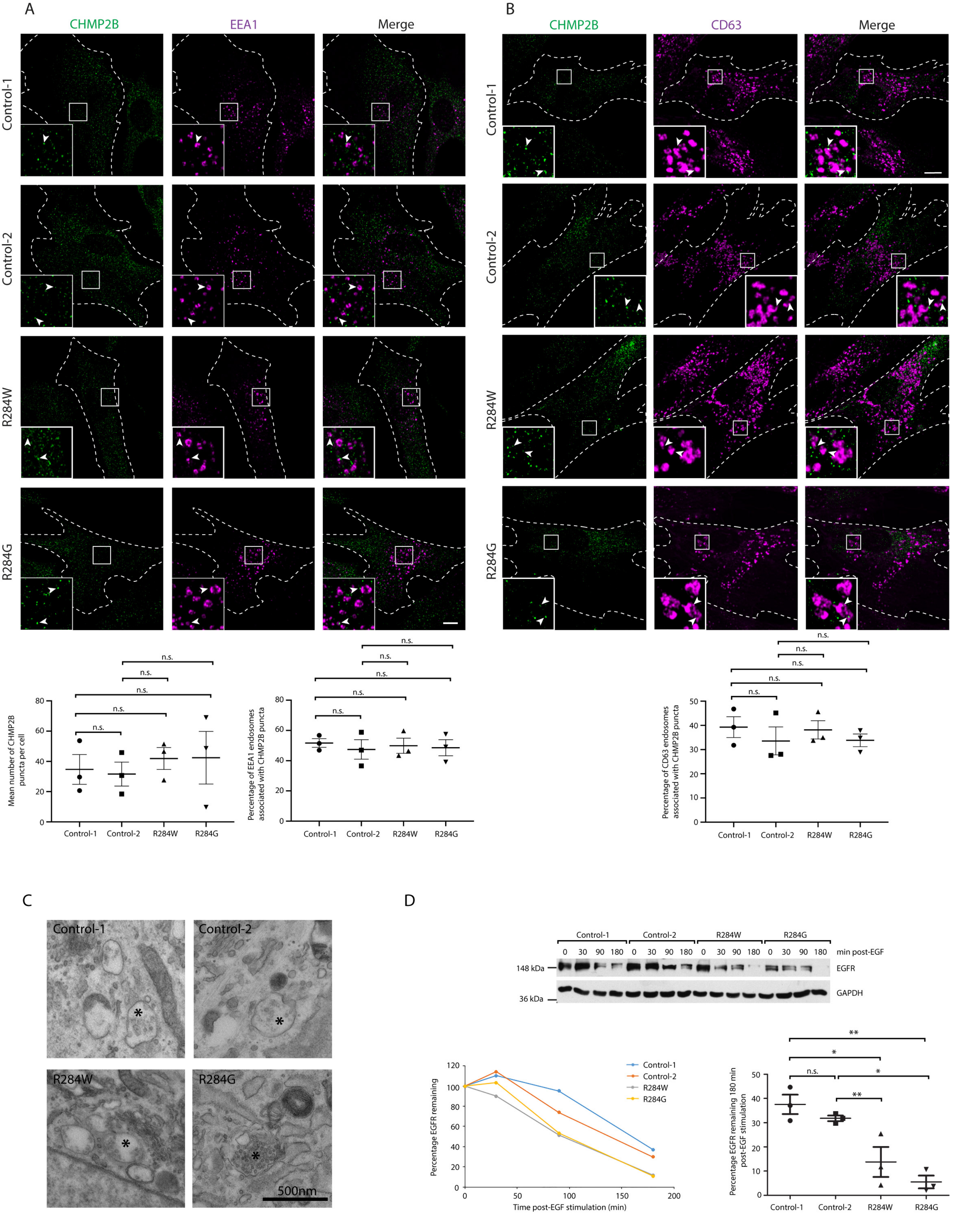
A
CHMP2B
EEA1
Merge
Control-1
Control-2
R284W
R284G
n.s.
Mean number of CHMP2B puncta per cell
Percentage of EEA1 endosomes associated with CHMP2B puncta
Control-1
Control-2
R284W
R284G
B
CHMP2B
CD63
Merge
Percentage of CD63 endosomes associated with CHMP2B puncta
C
Control-1
Control-2
R284W
R284G
500nm
D
Control-1
Control-2
R284W
R284G
0 30 90 180
min post-EGF
148 kDa
36 kDa
EGFR
GAPDH
Percentage EGFR remaining
Time post-EGF stimulation (min)
Percentage EGFR remaining 180 min post-EGF stimulation
n.s.
*
**

Figure S5

Figure S5. Proband fibroblasts show no defects in endosomal functions of the core ESCRT-III complex. A-B) Cultured fibroblasts from the control subjects or the probands indicated were fixed, labelled against EEA1 (**A**) or CD63 (**B**) and the core ESCRT-III complex member CHMP2B, then visualised by confocal immunofluorescence microscopy. In (**A**) the number of CHMP2B puncta and the percentage of EEA1-positive endosomes associated with a CHMP2B punctum was quantified in 3 experiments (5 cells per experimental condition in each repeat) and plotted in the graphs beneath the images. In (**B**) the percentage of CD63-positive endosomes associated with a CHMP2B punctum was quantified in 3 experiments (in 5 cells per experimental condition in each repeat) and plotted beneath the images. Micrograph scale bar = 10 μm. Arrows indicate juxtaposed or co-localised puncta. **C**) Fibroblasts derived from the subjects indicated were imaged for transmission electron microscopy. MVBs containing ILVs were evident in all cases (indicated by *). Scale bar = 500 nm. **D**) Fibroblasts from probands and controls were stimulated with EGF, lysed at the times indicated, then immunoblotted to monitor EGFR degradation. The line chart on the left shows EGFR quantification normalised to the initial amount of EGFR for a representative experiment, while the plot on the right shows the percentage of EGFR remaining at the 180 min time point for n=3 such experiments. In all plots, bars indicate mean ± S.E.M., p-values calculated by one-way ANOVA with Tukey's post-hoc test for repeated measures.

A

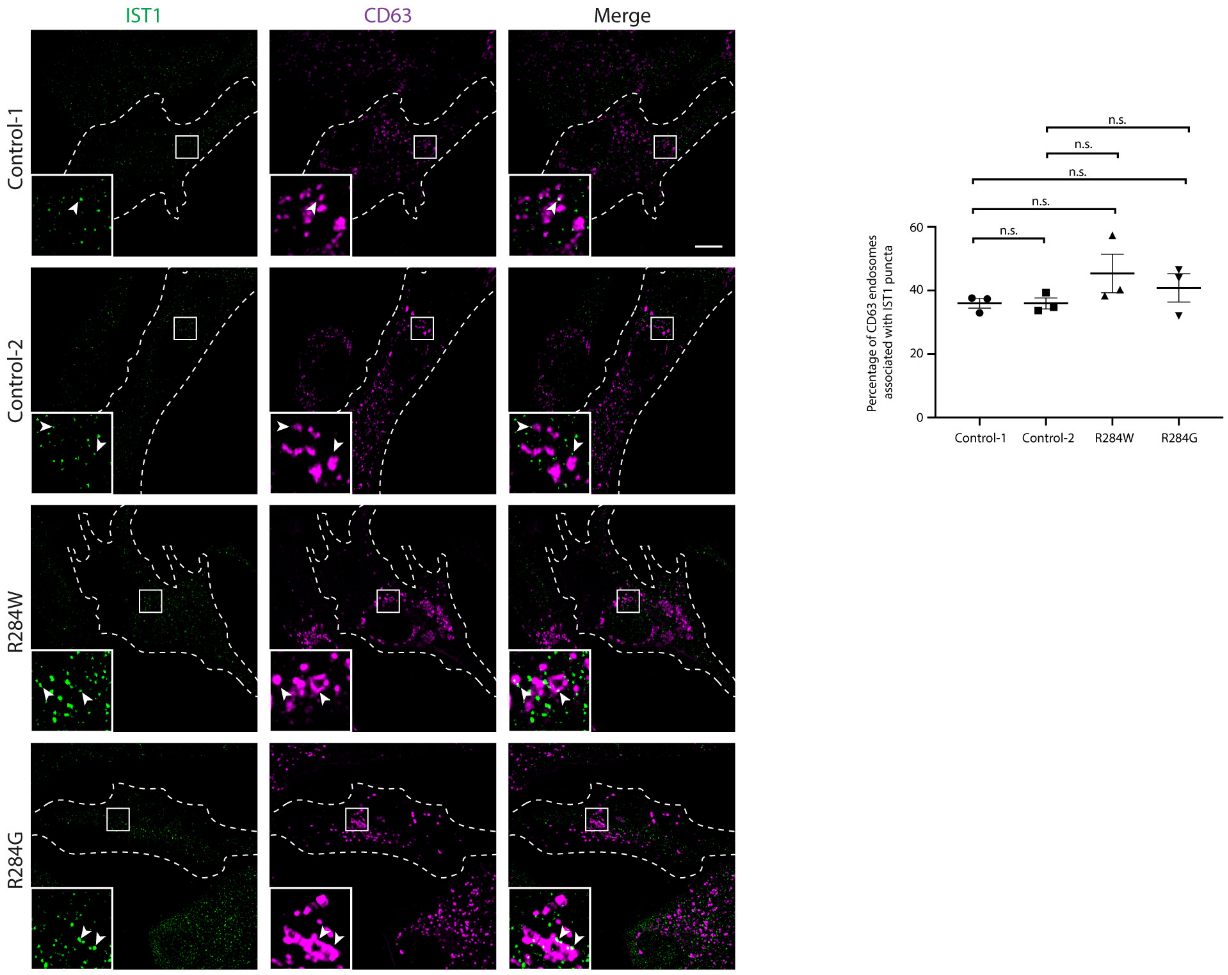
IST1
CD63
Merge
Control-1
Control-2
R284W
R284G
Percentage of CD63 endosomes associated with IST1 puncta
0
20
40
60
n.s.
n.s.
n.s.
n.s.
n.s.
Control-1
Control-2
R284W
R284G

B

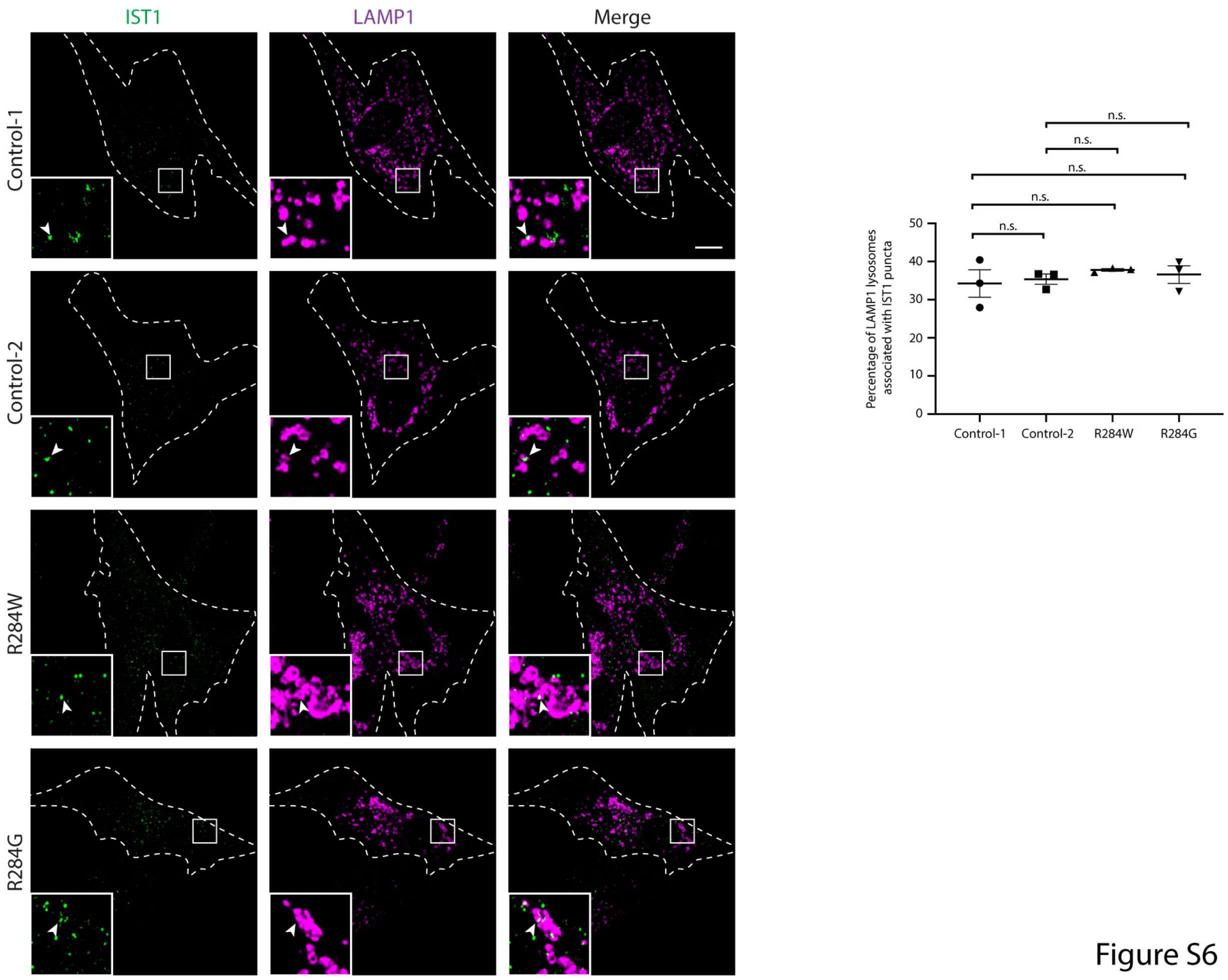
IST1
LAMP1
Merge
Control-1
Control-2
R284W
R284G
Percentage of LAMP1 lysosomes associated with IST1 puncta
0
10
20
30
40
50
n.s.
n.s.
n.s.
n.s.
n.s.
Control-1
Control-2
R284W
R284G

Figure S6

Figure S6. No increased recruitment of IST1 to late endosomes or lysosomes in proband fibroblasts. Cultured fibroblasts from the control subjects and the probands indicated were fixed and labelled for **A**) IST1 and CD63 or **B**) IST1 and LAMP1. The percentage of CD63 or LAMP1-positive endosomes associated with an IST1 punctum was quantified in 3 experiments per marker (in 5 cells per experimental condition in each repeat) and plotted in the corresponding charts. Arrows indicate juxtaposed or co-localised puncta. Bars indicate mean ± S.E.M., p-values were calculated by one-way ANOVA with Tukey's post-hoc test for repeated measures. Micrograph scale bar = 10 μm.

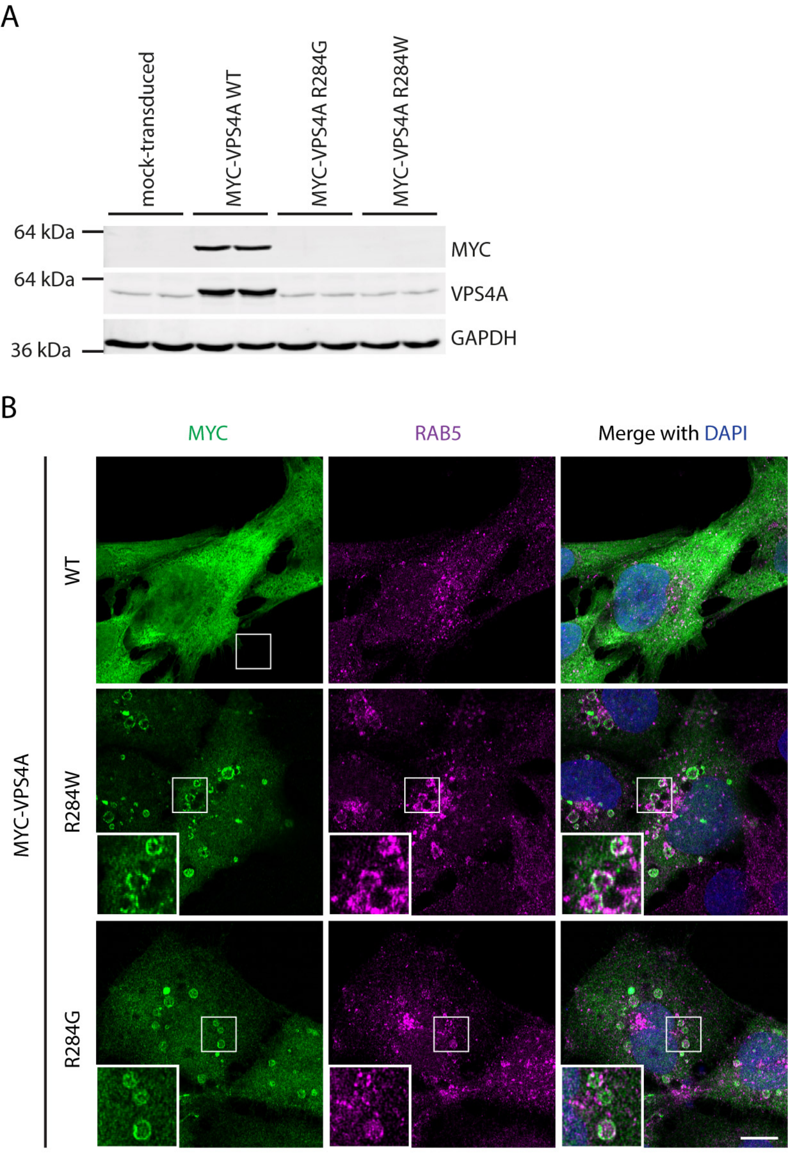
A
mock-transduced
MYC-VPS4A WT
MYC-VPS4A R284G
MYC-VPS4A R284W
64 kDa
64 kDa
36 kDa
MYC
VPS4A
GAPDH
B
MYC
RAB5
Merge with DAPI
MYC-VPS4A
WT
R284W
R284G

Figure S7

Figure S7. Expression of VPS4A containing disease-associated sequence changes causes the development of vacuolar endosomal structures in iPSCs. A) I^3 iPSCs were lentivirally transduced with expression constructs for myc-tagged wild-type VPS4A, or forms of VPS4A containing the p.R284G or p.R284W disease-associated changes. Cell lysates were then immunoblotted with the antibodies indicated. GAPDH immunoblotting serves as a control to verify equal protein loading in each lane. **B**) Transduced cells were also fixed and labelled with antibodies against the myc epitope and RAB5, then visualised by confocal immunofluorescence microscopy. Examples of rare cells containing large vacuolar endosomal structures are shown in the inset higher magnification boxes. Micrograph scale bar = 10 μm.

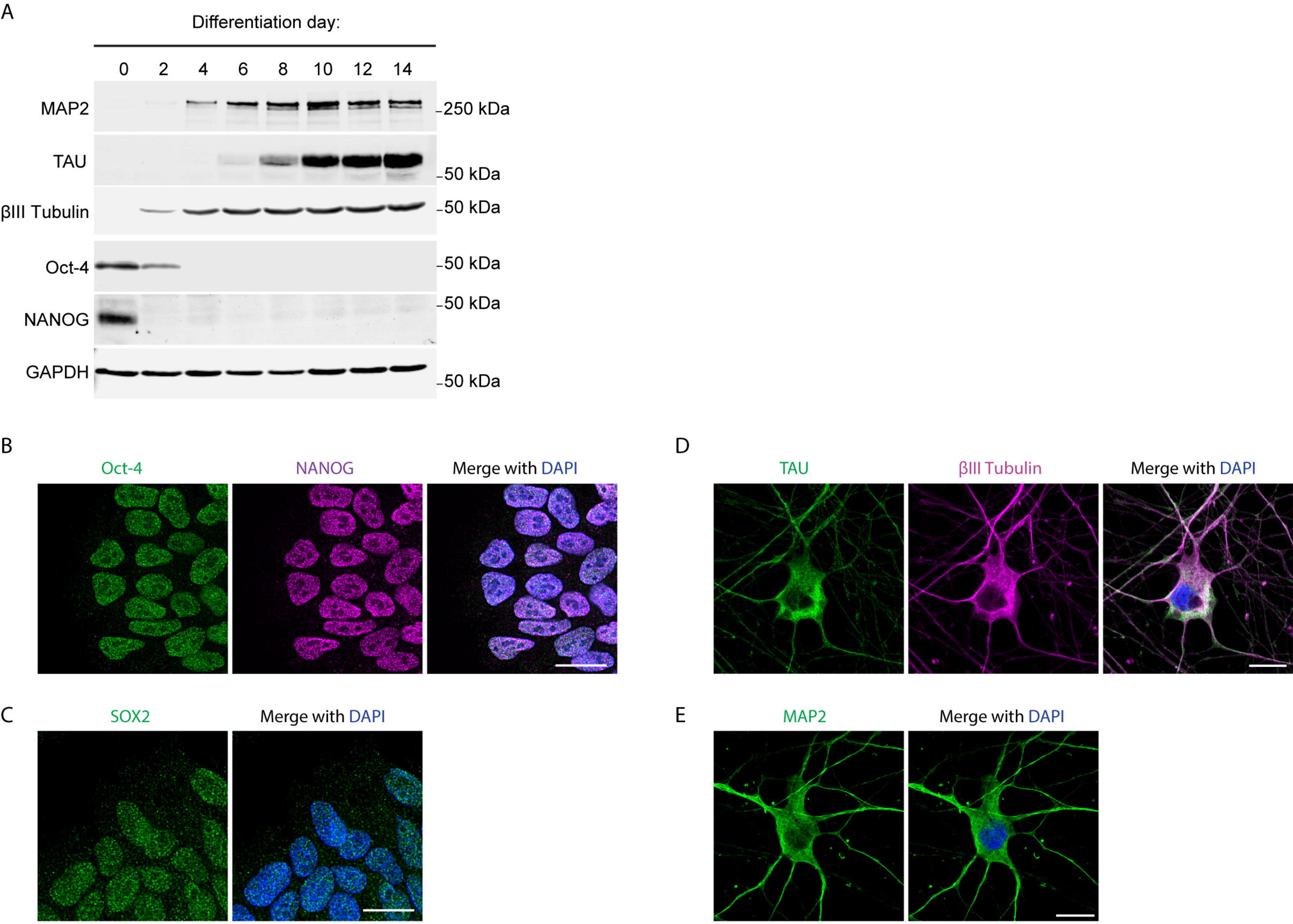
A
Differentiation day:
0 2 4 6 8 10 12 14
MAP2
TAU
βIII Tubulin
Oct-4
NANOG
GAPDH
250 kDa
50 kDa
50 kDa
50 kDa
50 kDa
50 kDa
B
Oct-4
NANOG
Merge with DAPI
C
SOX2
Merge with DAPI
D
TAU
βIII Tubulin
Merge with DAPI
E
MAP2
Merge with DAPI

Figure S8

Figure S8. Time course experiment demonstrating loss of pluripotency and gain of neuronal characteristics in i^{3}N iPSCs and neurons. A) i^{3}N iPSCs were cultured in the presence of doxycycline for 3 days. Cell lysates were then made at the time points indicated and blotted against the neuronal differentiation markers MAP2, Tau and βIII-tubulin, as well as the pluripotency markers Oct-4 and NANOG. GAPDH immunoblotting serves as a control to validate equal protein loading across lanes. **B-E**) i^{3}N iPSCs (**B** and **C)** and i^{3}Neurons 14 days post induction of differentiation (**D** and **E**) were fixed and processed for confocal immunofluorescence microscopy with the pluripotency (Oct-4, NANOG and SOX2) and neuronal markers (TAU and βIII-tubulin) indicated.

**Patient number**	Patient 1	Patient 2	Patient 3	Patient 4	Patient 5	Patient 6
Gender (M/F)	F	M	M	F	F	M
Ethnicity	Caucasian (White British)	Caucasian	Caucasian (White British)	Caucasian	Caucasian	Caucasian (White British)
**Genetic Infirmation**						
(Genome Build)	38	37	38	38		38
gDNA (Genome Build)	16:69320768 A>T	chr16:69354671 A>G	16:69320768 A>T	16:69320768 A>T	16:69320768 A>T	16:69319539G.A
cDNA	c.850A>T	c.850A>G	c.850A>T	c.850A>T	c.850A>T	c.616G>A
Protein (NM_013245.2)	p.Arg284Trp	p.Arg284Gly	p.Arg284Trp	p.Arg284Trp	p.Arg284Trp	p.Glu206Lys
Mutation type	missense	missense	missense	missense	missense	missense
REVEL score	0.97	0.983	0.97	0.97	0.97	0.917
CADD score	35	34	35	35	35	34
Inheritance	de novo	de novo	de novo	de novo	de novo	de novo
Analysis method	WGS	WES/WGS	WGS	WGS	WES	WGS
**Clinical Information**						
Age at last examination (y or m)	2 y	29 y	2 y	6 m	6 y	
Deceased (age)	no	yes (29 y)	yes (26 m)		no	no
Cause of death		respiratory failure complicating pneumonia	bowel ischaemia and volvulus			
**Neurologic**						
Intellectual disability (IQ or severity)	severe	severe	severe	severe	severe	severe
Developmental delay	severe	severe	severe	severe poor head control	severe	severe
Motor delay	yes	severe	severe		yes	yes
Speech delay	yes	severe	absent		yes	absent
Epilepsy	no	yes	no	yes	no	yes
Hypotonia	yes	yes	yes	yes	yes	no
Spasticity	mild in upper limbs	yes	yes		no	yes
Ataxia	yes	not assessed - severe motor delay	no		non-ambulatory; dysmetria	no
Dystonia	yes profound	yes	yes	yes	yes	no
Other abnormal movements	no	no	chorea		no	no
Sleep disturbances	yes	yes	unknown		yes; central sleep apnea	yes
**Brain imaging**						
Major features	hypoplastic cerebellar vermis and hemispheres	cerebellar hypoplasia	bilateral polymicrogyria and pontocerebellar hypoplasia	pontocerebellar hypoplasia	progressive pontocerebellar atrophy involving vermis and hemispheres;	severe cerebral atrophy on CT at 1 year
Other MRI findings	thin corpus callosum	corpus callosum hypolasia	follow - up MRI parenchymal loss and possible dysmyelination		decereased cerebral white matter volume with preiventriculat gliosis	
**Growth Parameters**						
Delivery (wks+days)	37	39	40	38+6	33	38+4
Weight at birth (gr; SD or centile)	2310 g (Z -2.8)	3120 g (25th)		2450 g (Z -2.4)	2083 g (50th-75th)	2073 g (<0.04th)
Length at birth (cm; centile)		48 cm (9-25th)			45 cm (75th)	
Head circumference at birth (cm; SD or centile)	31.4 cm (Z -2.6)	35 cm (50th)		30.5 cm (<0.04th, Z< -3)	25 cm (<0.04th, Z<-3)	31 cm (0.04th-2nd)
Microcephaly	yes	yes	yes	yes	yes	yes
Height (cm; SD)	74.5 cm (Z -1.87)	115 cm (Z -5.5)	78 cm (at 26m) (Z -3.0)		124 cm (Z 1.4)	
Head circumference (cm; SD or centile)		46 cm (Z -6.8)	42 cm (at 26m) (Z -4.5)	39 cm (at 6 m) (Z -5)	42.3 cm (<< 0.04th)	46 cm (Z -5.0)
Weight (kg; SD)	7.645 kg (Z -2.31)	15 kg (Z -6.0)	12.45 kg (at 26m) (Z -0.41)	6.56 kg (at 6 m) (Z -1.1)	21.3 kg (Z 0.17)	2nd-9th centiles
Feeding difficluties	yes - severe gastroesophageal reflux	yes	yes		yes - gastroesophageal reflux	no
Nasogastric (NG) feeding or percutaneous endoscopic gastrostomy (PEG)	NG/PEG-J	no (declined)	PEG/ fundoplication and jejunal tube		yes	no
**Eye phenotype**						
Congenital Cataract	yes	yes	yes		no	yes
Retinal Dystrophy	yes	no	salt and pepper retinitis		no	yes
Leber congenital amaurosis	no	no	no		yes	no
Vision	no fixing or following	poor fixing and no following	no fix and following	poor fixing and following at 6 m	diminished	diminished
Staphyloma		yes	no		no	
Aphakia	yes (post-operative)	no	no		no	yes
**Liver function**						
Hepatomegaly	yes and conjugated bilirubinaemia at 1 yr	mild hepatosplenomegaly	progressive hepatomegaly from 1 year of age		congenital hepatosplenomegaly	no
Other	raised AFP	no	haemosiderosis		mild cholestasis, iron overload secondary to CDA-1	
Gallstones	no	yes	microvesicular steatosis		no	no
**Musculoskeletal anomalies**						
Lipodystrophy	no	yes	no		no	no
Abnormal CPK	no	yes mild on 3 occasions	yes on 2 occasions		not done	no
Muscle biopsy	no	no	myopathic		no	no
Scoliosis	no	yes, severe	no		no	yes
Toe abnormalities		II toes hypoplasia	no	short toes	no	
Hip dysplasia	no	yes	no		coxa valga	yes
Single palmar creases	no	yes	no		no	no
Talipes (bilateral)	no	yes	no	yes	no	yes
**Haematological anomalies**						
Anemia	yes- raised reticulocyte count and platelets<br>blood film - anisopoikilocytosis	macrocytic anemia with anisopoikilocytosis	no		yes	no
Hemolytic crisis	no	yes	no		no	no
Bone marrow biopsy/aspiration	no	dyserythropoiesis	no - normal at post mortem			no
Congenital dyserythropoitic anaemia		yes	no		yes	no
**Other features**						
Renal defects	no	no	no			no
Liver fibrosis	no	no	no			no
Sensorineural deafness	no	yes	no		yes	no
Hypogonadism	no	yes	no			no
Stipsis	no	yes	yes		yes	no
Stomatitis	no	yes	no		no	no
Dental anomalies	no	yes	no			no (delayed eruption)
Recurrent infections	no	yes	no		no	no
Aphakia	post operative	no	no		no	yes
Other comments						left testicular torsion and right undescended testis

Table S1. Genetic and clinical features of patients with de novo heterozygous VPS4A sequence alterations.
